# Acute degradation of nucleolin reveals its novel functions in cell cycle progression and cell division in triple negative breast cancer

**DOI:** 10.1186/s13046-025-03401-y

**Published:** 2025-07-14

**Authors:** Joseph Mills, Anna Tessari, Vollter Anastas, Damu Sunilkumar, Nastaran Samadi Rad, Saranya Lamba, Ilaria Cosentini, Ashley Reers, Zirui Zhu, Wayne O. Miles, Vincenzo Coppola, Emanuele Cocucci, Thomas J. Magliery, Heather Shive, Alexander E. Davies, Lara Rizzotto, Carlo M. Croce, Dario Palmieri

**Affiliations:** 1 Department of Cancer Biology and Genetics, College of Medicine, The Ohio State University, Columbus, OH 43210 USA; 2 The Ohio State University Wexner Medical Center and Comprehensive Cancer Center, Columbus, OH 43210 USA; 3 Molecular, Cellular, and Developmental Biology Graduate Program, The Ohio State University, Columbus, OH 43210 USA; 4 Oncology Unit, AULSS5 Polesana, Rovigo, 45100 Italy; 5 Graduate School of Biomedical Sciences, Tufts University, Boston, MA 02155 USA; 6 Biomedical Sciences Graduate Program, The Ohio State University, Columbus, OH 43210 USA; 7 Current address: Institute for Biomedical Research and Innovation (IRIB), National Research Council of Italy (CNR), Palermo, Italy; 8 Current address: Department of Ecology and Evolutionary Biology, Tulane University, New Orleans, LA 70118 USA; 9 Department of Chemistry and Biochemistry, The Ohio State University, Columbus, OH 43210 USA; 10 Chemistry Graduate Program, The Ohio State University, Columbus, OH 43210 USA; 11 Pelotonia Institute for Immuno-Oncology, The Ohio State University-James Cancer Hospital and Solove Research Institute, Columbus, OH 43210 USA; 12 Division of Pharmaceutics and Pharmacology, College of Pharmacy, The Ohio State University, Columbus, OH 43210 USA; 13 Department of Veterinary Biosciences, College of Veterinary Medicine, The Ohio State University, Columbus, OH 43210 USA; 14 Current Address: Laboratory of Cancer Biology and Genetics, National Cancer Institute, National Institutes of Health, Bethesda, MD 20892 USA; 15 Current address: Division of Oncological Sciences, Department of Pediatrics, Cancer Early Detection Advanced Research Center, School of Medicine, Oregon Health and Science University, Portland, OR 97239 USA; 16 Gene Editing Shared Resource, The Ohio State University Wexner Medical Center and Comprehensive Cancer Center, 908 Biomedical Research Tower, 460 West 12th Avenue, Columbus, OH 43210 USA

**Keywords:** Nucleolin, NCL, Cell cycle, Auxin-inducible degron

## Abstract

**Introduction:**

Nucleoli are large nuclear sub-compartments where vital processes, such as ribosome assembly, take place. Most nucleolar proteins are essential; thus, their abrogation cannot be achieved through conventional approaches. This technical obstacle has limited our understanding of the biological functions of nucleolar proteins in cell homeostasis and cancer pathogenesis.

**Methods:**

We applied the Auxin Inducible Degron (AID) proteolytic system, paired with CRISPR/Cas9 knock-in gene-editing, to obtain an unprecedented characterization of the biological activities of Nucleolin (NCL), one of the most abundant nucleolar proteins, in Triple Negative Breast Cancer (TNBC) cells. Then, we combined live-cell imaging, RNA-sequencing, and quantitative proteomics, to characterize the impact of NCL acute abrogation on the behavior of TNBC cells. Finally, we used in silico analyses to validate NCL molecular role in TNBC patients.

**Results:**

Acute abrogation of endogenous NCL impacted both the transcriptome and the proteome of TNBC cells, particularly affecting critical players involved in ribosome biogenesis and in cell cycle progression. Unexpectedly, NCL depletion limited cancer cell ability to effectively complete cytokinesis, ultimately leading to the accumulation of bi-nucleated cells. In silico analyses confirmed that the levels of regulators of cell cycle progression and chromosome segregation correlated with NCL abundance in TNBC patients. Finally, NCL degradation enhanced the activity of pharmaceutical inhibitors of cellular mitosis, such as the Anaphase Promoting Complex inhibitor APCin.

**Conclusions:**

Our findings indicate a novel role for NCL in supporting the completion of the cell division in TNBC models, and that its abrogation could enhance the therapeutic activity of mitotic-progression inhibitors.

**Supplementary Information:**

The online version contains supplementary material available at 10.1186/s13046-025-03401-y.

## Introduction

The nucleolus is a prominent sub-nuclear organelle of eukaryotic cells [1]. The most extensively described function of the nucleolus is the synthesis of ribosomal RNA (rRNA) and assembly of the ribosomes [1, 2]. The nucleolus also acts as a sequestration compartment for proteins with critical cellular functions such as cell cycle progression, telomere elongation, DNA damage response, and cell death [3]. For these reasons, the nucleolus is now considered a central hub where multiple intra- and extra-cellular signals converge and modulate cell homeostasis and stress response [1, 3, 4].

Alterations of nucleolar size, structure, and number are associated with physiological aging and a wide range of human diseases, such as neurodegenerative disorders, progeria, and cancer [5–8]. Particularly in breast cancer, nucleolar area is associated with reduced disease-free survival after surgical tumor resection [9]. For these reasons, nucleolar histologic features are considered an underestimated, clinically relevant indicator associated with poor prognosis [9, 10].

In this study, we investigated the biological activities of nucleolin (NCL), one of the most abundant nucleolar proteins in human cells [11, 12]. NCL is involved in rRNA expression and maturation, chromatin remodeling, and translational regulation [12, 13]. NCL is also consistently upregulated in human tumors. However, deeper understanding of NCL biological functions has remained elusive, which has prevented the clinical translation of anti-NCL agents thus far [14–16].

It remains to be determined, for example, whether NCL overexpression has a causal role in cancer pathogenesis or is the result of the transformation process itself. This knowledge gap is partially due to experimental challenges in modulating the expression or the activity of nucleolar proteins in cellular systems. In fact, overexpression of NCL has proven difficult, due to its extreme abundance at the basal level [17]. On the other end, most nucleolar proteins have an essential role, therefore the use of knock-out or shRNA/siRNA approaches, all requiring several days to achieve their effect, is not ideal to assess their direct biological functions [18]. Finally, both overexpression and silencing of nucleolar proteins can lead to a chronic alteration of cell homeostasis, ultimately preventing the ability to discriminate between the biological functions of these proteins and the downstream consequences of their experimental alterations [19]. Overcoming these limitations would improve our understanding of the biological functions of nucleolar proteins, supporting the development of appropriate therapeutic approaches for human diseases where nucleolar functions are altered.

Here, we leveraged the Auxin Inducible Degron (AID) *“*on-demand*”* proteolytic system to push the boundaries imposed by NCL knock-down, knock-out, or overexpression approaches [20, 21]. Using CRISPR/Cas9-based gene editing, we generated Triple Negative Breast Cancer (TNBC) cell lines where endogenous NCL is fused to an AID tag, to promptly control its cellular levels using the phytohormone auxin with unprecedented specificity and temporal resolution. Then, we performed short-term functional assays to assess the biological impact of NCL acute depletion on the biology of TNBC cells and validated our results using in silico analyses of TNBC patients’ data. Overall, our findings support a novel role for NCL in the control of the cell division cycle in TNBC cells.

## Results

### NCL is upregulated in aggressive breast cancer and TNBC

**Fig. 1:**
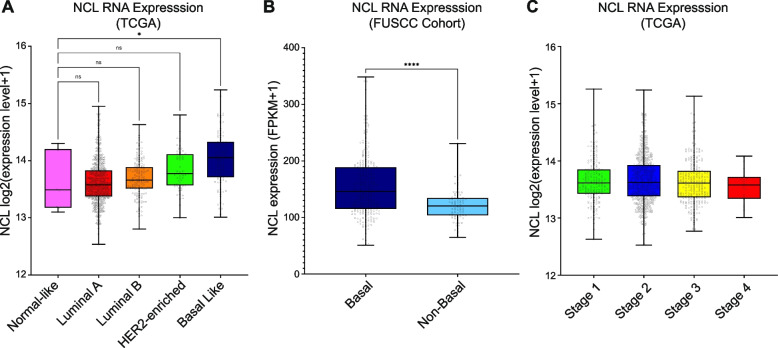
NCL is overexpressed in aggressive human breast tumors. **A** NCL RNA expression levels among different breast tumor subtypes compared with normal-like breast cancer samples. Data expression levels were obtained from 915 patient samples (Normal-like, *n* = 8; Luminal A, *n* = 624; Luminal B, *n* = 127; HER2-enriched, *n* = 58; Basal Like, *n* = 98) available through The Cancer Genome Atlas (TCGA). Significance was defined using Kruskal–Wallis test. **B** NCL RNA expression levels in Basal *versus* non-Basal TNBC patients. Data expression levels were obtained from 360 primary Chinese TNBCs (Basal, *n* = 277; Non-Basal, *n* = 83) available through the Fudan University Shanghai Cancer Center (FUSCC). Significance was defined using Mann–Whitney test. **C** NCL RNA expression levels among 1,076 breast tumor clinical stages, as described in A (Stage 1, *n* = 181; Stage 2, *n* = 624; Stage 3, *n* = 251; Stage4, *n* = 20). Significance was defined using Kruskal–Wallis test. Ns: not significant; *: *p* < 0.05; ****: *p* < 0.0001

NCL has been previously reported to be upregulated in most human tumors, particularly BC [14, 22]. However, a comprehensive analysis of NCL RNA and protein expression in different subtypes and stages of BC had not been performed. We examined patient data collected by The Cancer Genome Atlas (TCGA) [23], where breast cancer samples are subdivided into the following subtypes based on their gene expression profiling: Normal-like, Luminal A, Luminal B, HER2-enriched, and Basal-Like [24]. Our analysis revealed that *NCL* expression levels were significantly higher in the Basal-Like BC subtype, in comparison with Normal-like samples, than any other subtype (Fig. 1A). A similar analysis on protein data of the CPTAC database [25] confirmed that Basal-Like BC subtypes expressed the highest levels of NCL protein, in comparison with the others (Figure S1 A). We also observed a significant increase in NCL protein levels in Basal-Like BC samples in comparison with all the non-basal BC subtypes pooled together (Figure S1B).

The Basal-Like subtype of BC patients includes the vast majority of TNBC samples [26]. Therefore, we assessed *NCL* expression levels on an independent dataset (FUSCC cohort [26]) of TNBC patients, previously stratified as Basal (characterized by an aggressive phenotype and poor prognosis) or non-Basal (less aggressive and with a better prognosis). Our analysis indicated that NCL levels were significantly higher in the Basal subtype of TNBC (Fig. 1B). Finally, we queried the TCGA database to assess whether *NCL* overexpression was associated with specific clinical stages of BC development. BC samples classified as Stage I through IV did not display a significant association between NCL overexpression and BC clinical stage (Fig. 1C).

Altogether, these results indicate that, among all BCs, Basal-Like BCs, and specifically TNBC with worse prognosis and aggressive phenotype, display higher NCL RNA and protein levels. Notably, NCL expression did not change during clinical progression, suggesting that its overexpression might be associated with the early stages of BC pathogenesis.

### The auxin inducible degron system accomplishes an efficient and fast depletion of NCL

Since NCL is significantly upregulated in TNBC, we decided to investigate the effects of its complete abrogation in a cellular model of this BC subtype. We used the Cancer Dependency Map (DepMap) [27, 28] portal to identify the most suitable TNBC cell line for our study. NCL was considered a commonly essential gene both using RNAi-based systems (Figure S2 A) and CRISPR-based knock-out (Figure S2B), not only in BC cell lines, but in any available cell line. These observations strongly suggest that the generation of a cancer cellular system where NCL is completely and chronically abrogated is either not possible or would require significant adaptation phenomena for the cells to survive.

We reasoned that a targeted proteolysis system would be the best tool to achieve a rapid, “on-demand” degradation of NCL, and we decided to take advantage of the Auxin Inducible Degron (AID) system [20, 21] in combination with CRISPR/Cas9-based knock-in gene editing to modify the endogenous *NCL* gene.

The AID system has never been used for endogenous proteins with nucleolar localization to date, but it could allow the study NCL biological functions with unprecedented temporal resolution. As a cellular model, we chose MDA-MB-231, a widely used TNBC cell line, expressing high levels of endogenous NCL [22]. This specific cell line was also selected based on previous studies investigating the impact of NCL inhibition in breast cancer cell lines [22]. The absence of copy number variations (CNVs) of the *NCL* gene in this cell line was confirmed by the data available in the DepMap portal (Figure S2 C).

**Fig. 2:**
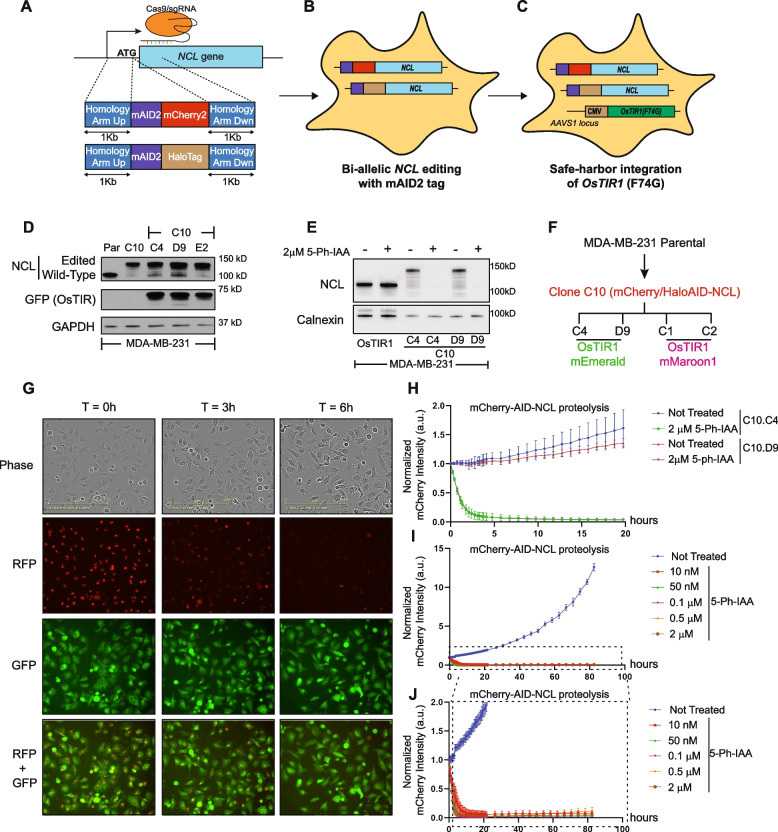
The Auxin Inducible Degron system accomplishes an efficient and fast depletion of NCL. **A**-**C** Schematic representation of the approach used for the generation of homozygous AID-NCL/OsTIR1 cell lines. Multi-allelic editing of endogenous *NCL* gene was achieved using two independent donor plasmids and a gRNA designed against the first translated codon of the gene (**A**). Selected multi-allelic *NCL* edited cells (**B**) were further modified to integrate the *OsTIR1* transgene into the *AAVS1*-safe harbor site (**C**). See also Figure S2. **D**. Western blot of endogenous NCL in engineered clones, in comparison with parental (Par) cells. Anti-GFP antibody was used to detect the OsTIR1 fused to mEmerald. GAPDH was used as loading control. **E** NCL degradation by western blot at 24 h upon 5-Ph-IAA treatment in two different MDA-MB-231 clones (C10.C4 and C10.D9) compared with MDA-MB-231 cells only transduced with OsTIR1. Calnexin was used as loading control.** F** Diagram of the hierarchical generation of the clones used in the current study. **G** Representative images of Incucyte experiments on MDA-MB-231 mCherry2/Halo-AID-NCL/OsTIR1-mEmerald cells. The RFP channel was used to detect mCherry2-AID-NCL, and the GFP channel was used to assess OsTIR1-mEmerald expression and localization. **H** Quantitative analysis of mCherry (AID-NCL) intensity from Incucyte experiments on the indicated cell clones. RFP integrated intensity per image was normalized for t = 42’ to account for potential plate condensation at the early time points. The experiment was performed in 5 biological replicates, with 5 technical replicates. Error bars show standard deviation. **I**-**J** Dose–response curve (**I**) and relative magnification (**J**) for mCherry-AID-NCL degradation using the indicated amounts of 5-Ph-IAA. RFP integrated intensity per image was normalized for t = 0. The experiment was performed in three biological replicates, with four technical replicates. Error bars show standard deviation

We employed a two step-editing CRISPR/Cas9-based knock-in approach to generate cells where endogenous NCL protein could be quickly degraded on demand. For the targeted genomic modification of the endogenous NCL, we engineered a plasmid containing two 1-Kb homology arms corresponding to the genomic region of the 5’-end of the endogenous *NCL* coding sequence (Fig. 2A). Notably, previous reports indicated that modifications of NCL N-terminal region does not affect its biological functions [29]. We included in this plasmid the mAID2 tag [21], in frame with either the mCherry2 [30] or the HaloTag [31] proteins. These two plasmids were used as donors for Cas9-based homology-driven repair (HDR) of the endogenous *NCL* gene. We decided to use two different constructs for gene-editing purposes because this strategy offers multiple advantages: first, it leads to the expression of endogenous NCL in frame either with the two fluorescent modules, allowing the tracking of its expression and localization; second, it allows for the selection of cells displaying a multi-allelic *NCL* editing, based on the presence of both fluorescent tags (Fig. 2B).

Following successful targeting, we then transduced the homozygous AID-NCL cells with a construct containing the *OsTIR1*, constitutively integrated in the *AAVS1* safe-harbor locus [32]. Specifically, we generated transgenic cell lines expressing OsTIR1(F74G) (a.k.a. OsTIR1v2), a point-mutant variant of the *Oryza Sativa* TIR1, whose activity is regulated by the auxin synthetic derivative molecule 5-phenyl-Auxin (5-Ph-IAA) [21].

We generated cells expressing OsTIR1(F74G) fused to the bright green fluorescent protein mEmerald [33] (Fig. 2C and S2D). Western blot analyses confirmed that gene-edited MDA-MB-231 (Clone C10) expressed the AID-NCL fusions. Moreover, C10 subclones (C10.C4, C10.D9, and C10.E2) also expressed OsTIR1v2 (detected using an anti-GFP/mEmerald antibody) (Fig. 2D). As expected, treatment with 2 µM 5-Ph-IAA for 24 h completely abrogated NCL to almost undetectable levels in both clones (Fig. 2E). This result also confirmed that the lower molecular weight bands detected by WB analyses correspond to AID-tagged NCL, potentially due to proteolysis or presence of post-translational modifications [34], as their presence was completely abrogated upon 5-Ph-IAA treatment.

To generate a novel AID system, compatible with other green channel-based experimental techniques, we generated an integration construct which co-expresses OsTIR1(F74G) and a histone H1.0 fluorescently tagged with the far-red protein mMaroon1 [35] (Figure S2E).

We obtained two additional independent clones (C10.C1 and C10.C2, summarized in Fig. 2F), where we confirmed OsTIR1 expression (Figure S2 F) and acute NCL degradation (Figure S2G).

NCL abrogation was also observed by live-cell imaging experiments (Fig. 2G and S2H). We quantified the total integrated RFP signal (corresponding to the AID-mCherry2-NCL allele) over time up to 20 h, confirming NCL abrogation at approximately 6 h of 5-Ph-IAA treatment in all the analyzed clones (Fig. 2H and S2I). We also observed that OsTIR-mediated AID-mCherry2-NCL degradation was only minimally dependent on the 5-Ph-IAA dose (Fig. 2I-J), and persisted up to 80 h. Live-cell imaging of OsTIR1-mEmerald (GFP channel) revealed enhanced localization of the enzyme at the nucleoli following 5-Ph-IAA treatment (Fig. 2G and S2H). However, in untreated cells, the enzyme had a homogeneous distribution in the cell, as previously described [33].

Altogether, these data demonstrate the effectiveness of the AID system in quickly degrading endogenously edited NCL.

### The acute abrogation of NCL reduces cell proliferation and alters the RNA levels of genes involved in cell cycle progression

Previous reports have shown contrasting results with accumulation of cells either in G1 or G2/M phase of the cell cycle after NCL silencing [18, 36]. We hypothesize that this discrepancy could be due to the limited efficacy and temporal resolution of the silencing system used in these studies. Therefore, we decided to test whether acute abrogation using our AID system could elucidate the role of NCL in cell cycle progression.

**Fig. 3:**
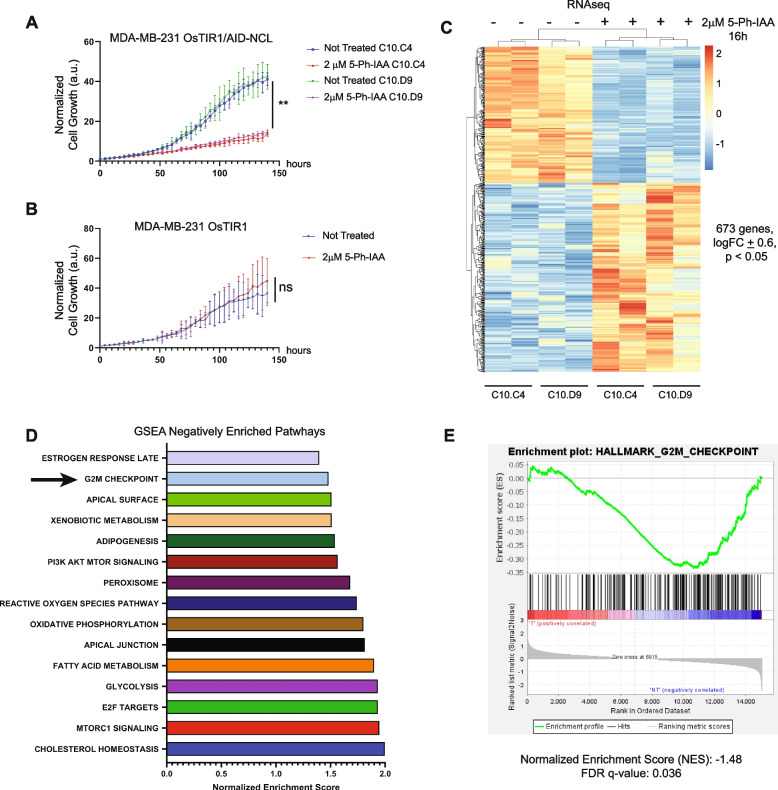
NCL acute abrogation alters the gene expression profile and proliferation of TNBC cells. **A**-**B** Growth curve of MDA-MB-231 OsTIR1/AID-NCL or control MDA-MB-231 OsTIR1 clones treated with 2 mM 5-Ph-IAA or left untreated for up to 140 h. Incucyte live-cell imaging system was used to monitor cell proliferation. The experiment was performed in five biological replicates, with 5 technical replicates. Statistical significance was calculated using 2-way ANOVA with Tukey’s multiple comparison test. Error bars show standard deviation. **: *p* < 0.01; ns: not significant. **C** Heatmap of RNA sequencing analyses on NCL-edited clones at 16 h upon 2 μM 5-Ph-IAA treatment or left untreated. Two independent clones in biological duplicate were used for the experiment.** D** Gene set enrichment analysis (GSEA) of Hallmark Pathways using RNA sequencing data of 5-Ph-IAA treated versus non-treated NCL-degron clones. Ranking of Normalized Enrichment Scores of the 15 most negatively enriched pathways after NCL abrogation. **E** Enrichment plot for the G2/M Checkpoint pathway, as described in **D**

NCL abrogation resulted in a marked reduction in proliferation, but only at 48 h from the treatment (Fig. 3A, Figure S3). Conversely, all control conditions (Fig. 3B, Figure S3) confirmed that neither 5-Ph-IAA treatment, nor NCL editing with the AID tag, nor OsTIR1 expression had any statistically significant effect on cell proliferation.

Since NCL is an RNA-binding protein [11], we decided to investigate whether the effect of NCL abrogation on cell proliferation could be due to alteration of RNA abundance, via RNA-sequencing. We analyzed total RNA from MDA-MB-231 AID-NCL cells treated with 2 µM 5-Ph-IAA for 16 h. This time point was selected to maximize NCL degradation while minimizing potential indirect effects on RNA abundance. As shown in Fig. 3C and in Supplementary Extended Data 1, 673 genes were significantly de-regulated upon NCL abrogation, including 390 genes upregulated and 283 genes downregulated (*p* < 0.05, LogFC ± 0.6). This relatively low number of genes is consistent with the short-term duration of this experiment, and potentially includes genes that are immediately associated with a direct regulation by NCL. Using Gene Set Enrichment Analysis (GSEA), we identified genes involved in G2/M cell cycle checkpoint among the most de-regulated pathways (Fig. 3D-E, Normalized Enrichment Score: −1.48; FDR q value: 0.036, see also Supplementary Extended Data 2).

These results indicate that acute NCL depletion has a rapid and negative effect on the expression of genes regulating cell proliferation.

### NCL abrogation causes cytokinesis defects

We wanted to determine which phase of the cell cycle was most impacted by NCL acute abrogation, ultimately resulting in reduced cell proliferation. We treated MDA-MB-231 AID-NCL/OsTIR1/H1.0-mMaroon1 cells with 5-Ph-IAA and analyzed their DNA content and levels of phospho-histone H3(S10), a known marker of mitosis (M phase) [37].

**Fig. 4:**
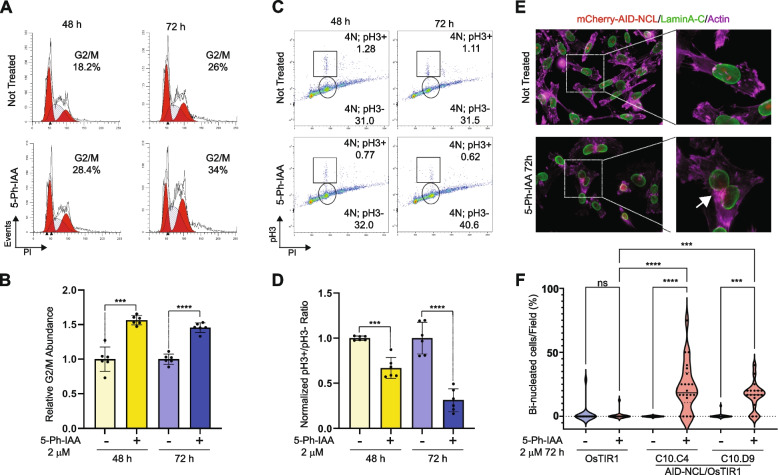
NCL acute abrogation induces defects of cell cycle progression and cytokinesis. **A** Representative flow cytometry data of cell cycle analyses by propidium iodide staining of OsTIR1/AID-NCL-edited MDA-MB-231 clones. The percentage of cells in each phase of the cell cycle was obtained by curve deconvolution using ModFit. The absolute abundance of cells in G2/M phase for the specific representative images is also reported. **B** Quantitative analyses of the relative percentage of cells in G2/M phase, as shown in A. Data are representative of two independent clones analyzed in three experiments performed in technical duplicate and normalized for the percentage of cells in G2/M in untreated controls. Significance was calculated using Welch’s t-test. **C** Representative flow cytometry data of propidium iodide/phospho-Histone H3 (S10) staining in cells. Gating was designed to quantitate the absolute population of cells with a 4 N content of DNA, and either positively (4 N; pH3 +) or negatively (4 N; pH3-) stained for pH3(S10) antibody. **D** Quantitative analyses of the 4 N; pH3 +/4 N; pH3- abundance ratio, as shown in C. Data are representative of two independent clones analyzed in three experiments performed in technical duplicate and normalized for the untreated controls. Significance was calculated using Welch’s t-test. **E** Representative images of immunofluorescence images of AID-NCL edited MDA-MB-231 clones after the treatment with 2 μM 5-Ph-IAA for 72 h or left untreated. LaminA/C staining (green) was used to detect nuclear membrane. Phalloidin-iFluor 647 staining (magenta) was used to stain actin cytoskeleton membrane. Edited mCherry2-AID-NCL is also visible in untreated cells (red). White arrow indicates actin patches in a bi-nucleated cell. **F** Quantification of bi-nucleated cells identified in the experiments shown in E. For each sample, at least 15 images, representative of independent fields, were taken on a single focal plane at 60 × magnification. A total of at least 100 cells were captured for each sample. The percentage of bi-nucleated cells per image was used to generate the violin plots. Statistical significance was calculated using one-way ANOVA with Sidak’s multiple comparison test. Data are representative of one of two independent experiments. **: *p* < 0.01; ***: *p* < 0.001; ****: *p* < 0.0001; ns: not significant

A significant increase in cells with a tetraploid (4 N) amount of DNA was observed at 48 and 72 h after NCL abrogation (Fig. 4A-B), indicating a potential G2/M arrest. In contrast, the populations corresponding to G1 (2 N) and S (< 4 N; > 2 N) phases of the cell cycle were only modestly, although significantly, affected by NCL depletion, except for 2 N cells at 72 h (Figure S4 A-B). However, we did not see a proportional increase in phospho-Histone H3(S10) (Fig. 4C-D), but rather a decrease in this population. These findings indicate that NCL abrogation does not cause prolonged arrest in the active phase of mitosis. These results are consistent with two possibilities: either NCL-depleted cells would accumulate in the G2 phase, at the end of the complete replication of DNA (S/G2 transition phase) but before the transition into M phase; or, in the absence of NCL, cells could accumulate in G1, but with a tetraploid content of DNA. To distinguish between these alternative hypotheses, we performed immunofluorescence staining of LaminA/C (a marker of nuclear lamina), and actin filaments (using Alexa-647 Phalloidin) to identify cell boundaries, after 72 h of 5-Ph-IAA treatment. NCL abrogation caused a significant increase in bi-nucleated cells in both clones, but not in control cells containing OsTIR1 and wild-type NCL, upon 5-Ph-IAA treatment (Fig. 4E-F). These results suggest that the increase in 4 N cells observed in Fig. 4A could be due to the accumulation of bi-nucleated cells.

Our findings were also confirmed by time-lapse microscopy imaging of 5-Ph-IAA-treated MDA-MB-231 AID-NCL/OsTIR1/H1.0-mMaroon1 cells (Supplementary Video 1). Interestingly, real-time imaging also showed that, upon NCL degradation, cells might undergo an asymmetrical 3-way cell division, ultimately leading to the formation of one mono- and one bi-nucleated cell.

Collectively, these results show that the acute abrogation of NCL impairs cell cycle progression and causes an accumulation of tetraploid, bi-nucleated cells.

### NCL expression is associated with proteins involved in chromosome segregation

We speculated that the increase in tetraploid cells could be due to defects of physical cell separation of the two daughter cells at the end of mitosis, defined as cytokinesis failure (reviewed in [38]). The best characterized causes of cytokinesis failure are: 1) defects/mutations of cytokinesis regulators; 2) long delays in mitosis (mitotic slippage); and 3) physical obstructions preventing the furrow cleavage after mitosis [38]. The transcriptome from NCL-depleted cells pointed toward mitotic slippage as the probable cause of cytokinesis failure, due to the reduced expression of genes involved in G2/M transition (Fig. 2A-C). However, delayed mitosis usually results in the formation of cells with a single tetraploid nucleus, or multiple micronuclei, which we did not observe in our TNBC NCL-depleted cells (Fig. 4). Furthermore, binucleated cells showed the accumulation of actin patches (Fig. 4E, right bottom panel), which have been previously shown as the result of the formation of chromosomal bridges and lagging chromosomes [39].

**Fig. 5:**
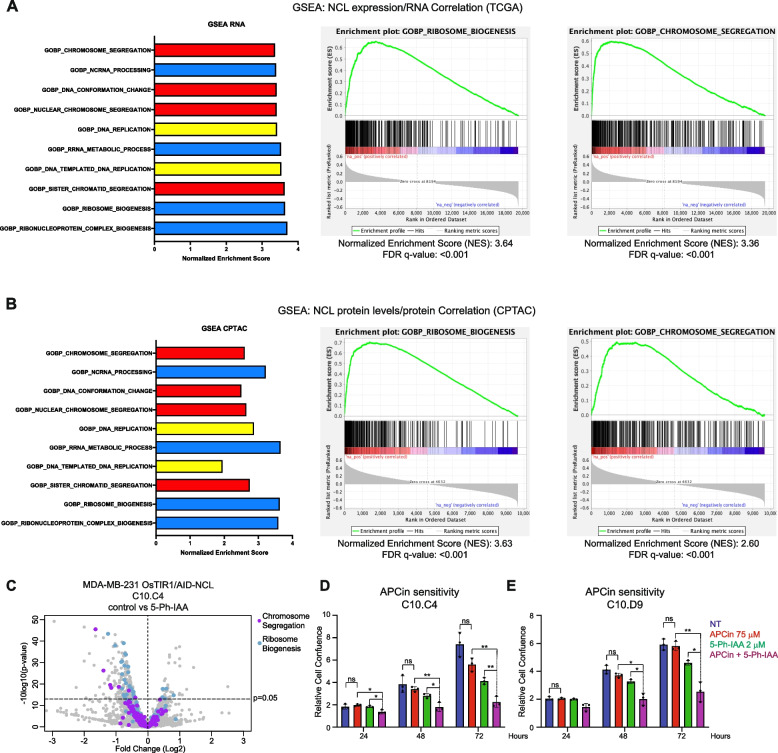
NCL levels are associated with altered sister chromatid segregation and sensitivity to inhibition of the Anaphase Promoting Complex. **A** Gene set enrichment analysis of GO Terms by gene ranking based on their correlation with NCL mRNA levels in the TCGA database. The top ten most enriched pathways positively correlated with NCL RNA expression are represented. Enrichment plots of two representative GO Terms are also reported (see also Figure S5). **B** Gene set enrichment analysis of GO Terms identified in A were analyzed based on their correlation with NCL protein levels in the CPTAC database. Enrichment plots of two representative GO Terms (as in A) are also reported. **C** Volcano plot of differentially abundant proteins upon NCL abrogation in MDA-MB-231 OsTIR1/AID-NCL cells (clone C10.C4), detected by TMT-proteomic analysis. Reported data excludes proteins with −10log_10_(*p*-value) > 50, for better visualization. For the full range, see Figure S5. Proteins included in the GO Terms “Chromosome Segregation” and “Ribosome Biogenesis” are reported in purple and blue, respectively. **D**-**E** Assessment of cell proliferation of two independent MDA-MB-231 OsTIR/AID-NCL clones, left untreated or treated with indicated doses 5-phIAA, APCin, or combination of both, for the indicated time points. Growth was assessed by Incucyte live-cell imaging and reported as relative growth normalized vs t = 36’. See also Figure S5. The experiment was performed in three biological replicates, with five technical replicates. Error bars show standard deviation. Statistical significance was calculated using 2-way ANOVA with Sidak’s multiple comparison test. Ns: not significant; *: *p* < 0.05; **: *p* < 0.01

Therefore, we hypothesized that higher NCL levels could be associated with increased levels of proteins involved in chromosomal dynamics in TNBC. To validate this hypothesis, we analyzed the TCGA database (Harvard Firehose Initiative) to reveal the transcripts positively correlating with NCL expression. The Spearman’s Correlation level for each gene was used to generate an ordered gene rank list, further analyzed by GSEA (Fig. 5A and S5 A). As expected, based on the known NCL roles in these processes [12, 34], the most enriched GO terms were “Ribosome Biogenesis” and “Ribonucleoprotein Complex Biogenesis”. However, five out of ten of the most enriched pathways included DNA dynamics processes, such as “Chromosome Segregation” and “Sister Chromatid Segregation”. Encouraged by these results, to assess whether NCL expression positively correlated with genes involved in DNA dynamics, we performed a parallel analysis of publicly available CPTAC proteomics data. All the ten pathways identified in the correlative analysis of the transcriptome were also highly correlating at protein level with the expression of NCL protein (Fig. 5B).

Then, we decided to assess in our TNBC in vitro model whether the acute depletion of NCL had any negative impact on the abundance of the proteins identified in silico. To this aim, we performed a Tandem Mass Tag quantitative proteomics analysis on MDA-MB-231 AID-NCL/OsTIR1 cells at 24 h after 5-Ph-IAA treatment to assess the global protein de-regulation upon NCL acute degradation (Extended Data 3). A general downregulation of proteins involved in Ribosome Biogenesis was observed (Figure S5B, Fig. 5C, blue dots, and Supplementary Table 1). Additionally, seven proteins involved in Chromosome Segregation (Fig. 5C, purple dots, and Supplementary Table 2) were significantly downregulated (*p* < 0.05) after NCL degradation, while no member of this GO term family was upregulated.

These results confirmed that depletion of NCL had a negative impact on factors involved in chromosomal segregation, potentially explaining the observed defects on cell cycle progression (Figs. 3 and 4).

Chromosomal segregation is regulated by the activity of the Anaphase Promoting Complex/Cyclosome (APC/C) and its regulatory subunit Cdc20 [40]. Inhibition of APC/C^Cdc20^ is synthetically lethal with defective sister chromatid cohesion [40]. Therefore, we hypothesized that altered sister chromatid dynamics upon NCL abrogation could enhance the activity of APC/C^Cdc20^ inhibitors, such as APCin [40]. To test this, we performed growth curves on MDA-MB-231 NCL-AID/OsTIR1 cells in the presence of 5-Ph-IAA, APCin, or a combination of the drugs. Figure 5D-E and S5 C-D show that APCin alone had no statistically significant effect on cell proliferation. In contrast, the simultaneous treatment with APCin and 5-Ph-IAA caused a significant reduction in cell proliferation, in comparison with either 5-Ph-IAA or APCin alone.

In summary, our data suggest that NCL abundance directly correlates with the levels of components of the chromosomal segregation machinery in TNBC patient samples and in our in vitro cellular model. Therefore, NCL abrogation has an enhanced anti-proliferative effect in combination with APC/C^Cdc20^ inhibitors, at least in part by impairing DNA dynamics.

## Discussion

Our understanding of nucleolar structural and biological properties has been propelled by the advancement of experimental tools in molecular and cellular biology. However, technical challenges, related to the experimental modulation of their expression, still prevent the molecular characterization of the biological functions of nucleolar proteins.

NCL is one of the most abundant nucleolar proteins [11, 12, 14]. In the present study, we provide in silico evidence that NCL overexpression occurs in a broad spectrum of breast tumors, with a significant upregulation in the most aggressive forms of BC, such as TNBC, both at the RNA and protein level (Fig. 1 and S1).

Like other nucleolar proteins, NCL biological functions remain poorly characterized, due to the lethal effect associated with its chronic downregulation. The NCL-AID system described here allows the study of endogenous NCL biological functions with unprecedented temporal resolution, before detectable adaptation or cell death can occur. This degradation kinetic is still slower than previously reported targets of the AID system (6 h *vs* 30–60 min) [20, 21]. It is conceivable that the reduced accessibility of nucleolar NCL to OsTIR1 could slow the pace of degradation in comparison with previous studies for non-nucleolar proteins.

We assessed the immediate (< 24 h) impact of NCL acute abrogation on cellular RNA abundance and observed a negative enrichment of genes involved in cell cycle progression, and specifically G2/M transition, resulting in impaired cell proliferation upon NCL abrogation (Fig. 3C-E). Previous studies, using RNAi-based NCL silencing systems, have reported contradictory results about the role of NCL in the regulation of the cell cycle [18, 41]. Our analyses showed that NCL acute depletion causes a marked increase in cells with a tetraploid content of DNA (4 N), but a decrease in phospho-Histone H3 + cells suggesting that this phenotype was not due to an increase in mitotic cells (Fig. 4A-D). Moreover, growth curve analyses showed that the effect on cell proliferation was detectable only after 48 and 72 h from NCL abrogation (Fig. 3A and S3). We speculated that the discrepancy between NCL degradation and the impact on cell proliferation could be due to a previously unreported role for this protein in regulating the abundance of proteins involved in cell cycle progression at the latest stages of cell division, rather than to a direct positive regulation of the cell division cycle entry. In line with this hypothesis, we found that NCL degradation results in a significant accumulation of bi-nucleated tetraploid cells containing remnants (actin patches) of failed cytokinetic processes (Fig. 4E-F).

It has been previously reported that tetraploid cells can attempt further cell division cycles, but the presence of multiple centrosomes leads to the formation of altered mitotic spindles [42]. Alterations of centrosome numbers and defective mitotic spindle assembly upon NCL silencing have been previously shown [18]. Therefore, we posit that the observed cytokinesis defects identified in the present study could represent the initial stage of the altered cellular phenotype reported earlier and indicate that the acute degradation mediated by our AID system allows a faster temporal characterization of NCL biological functions. These conclusions are further supported by the real-time observation that NCL-depleted cells can display non-symmetrical divisions leading to mono- and bi-nucleated cells.

Consistent with TCGA and CPTAC in silico analyses, we showed a significant reduction of proteins regulating chromosomal separation and cohesion in our in vitro model. This, in turn, leads to a significant increase in sensitivity to inhibitors of the Anaphase Promoting Complex/Cdc20 such as APCin, for NCL-depleted, chromosomal segregation-defective cells, as expected based on previous reports [40]. In fact, these compounds have previously shown their synthetic lethality with defects of chromatid separation or cohesion [40]. However, our data do not indicate that NCL exerts its biological function through the severe de-regulation of specific factors involved in the cell division machinery, as the magnitude of protein levels in our experimental model widely varied among individual components (Extended Data 3). This is not surprising, as cell division and cytokinesis are finely tuned, multi-component processes, whose efficiency can be positively or negatively modulated through orchestrated control mechanisms. Therefore, our data presented here suggest that NCL might be implicated in the overall positive tuning of cell division cycle.

Our findings support a role for NCL in regulating proteins that modulate cell cycle progression, chromosomal dynamics and segregation. However, the use of a TNBC cell line displaying dominant-negative mutations of *TP53* warrants the need of additional validation in multiple cell lines, including normal-like cells. Future studies will be required to further characterize the mechanisms through which NCL modulates these biological processes.

## Conclusions

 Here, we validate the AID system as a tool for the acute degradation of nucleolar proteins, which can provide a valuable resource for the study of their biological functions. This system could be used to investigate broader questions about the biology of NCL and of the nucleolus. For example, it can be used to study changes in biophysical properties depending on NCL abundance, or to assess the impact on the biogenesis of rRNA and ribosomes, or to measure the stability of proteins and mRNAs involved in cellular homeostasis during different phases of the cell cycle. Finally, the knowledge gained by this study can inform the development of new therapeutic strategies for TNBC, and potentially for a wide range of human tumors where NCL is over-expressed.

## Materials and methods

### Cell culture, transfections, and gene editing

MDA-MB-231 cell line was purchased from the American Type Culture Collection (ATCC HTB-26) and cultured in RPMI-1640 medium (Millipore Sigma) supplemented with 10% FBS (Millipore Sigma). Cells were grown in a humidified 37 °C incubator with 5% CO_2_. Identity of cell lines was validated by STR profiling. Cells were regularly tested for Mycoplasma contamination using MycoStripTM Mycoplasma Detection Kit (InvivoGen, #rep-mysnc-10).

To generate NCL-edited cells, 3 × 10^5^ cells were plated in a six-well plate transfected with the indicated gRNAs and donor plasmids (described above) using Lipofectamine™ 3000 Transfection Reagent (Thermo Fisher Scientific, #L3000001) in Opti-MEM™ I Reduced Serum Medium. (Thermo Fisher Scientific, #31985070). Cells were then grown to a subconfluent T175 cm^2^ flask in medium supplemented with 10% FBS. After incubation with 646-Janelia Fluor® HaloTag® Ligand (Promega, #GA1120), cells were sorted by FACSAria III for both mCherry positive fluorescence at 587 nm excitation, and HaloTag® far red 646 nm positive fluorescence. MDA-MB-231 parental cells and non-stained MDA-MB-231 cells were used as negative controls for mCherry and 646 fluorescence to design the gates. Double positive cells were collected in a 12-well plate and grown as a bulk population until sub-confluence in a T175 cm^2^ was reached. This bulk double-positive population underwent another round of sorting by FACSAria III to collect single cells in 96-well plates. Cells were then screened by fluorescence and western blot analysis for the selection of clones with homozygous expression of AID-NCL edited protein.

To generate NCL-edited cell lines constitutively expressing OsTIR1(F74G), 3 × 10^5^ AID-NCL edited cells (clone C10) were plated in a 6-well plate and transfected 24 later with donor plasmids ROLECCS V2 AS or OsTIR1(F74G)-IRES-H1-mMaroon, in combination with a plasmid expressing *AAVS1* safe harbor-specific gRNAs. Isolation and validation of single clones were performed as described above. A schematic hierarchy of the established cells lines used in the study is reported in Figure S2 F.

### Treatments

To induce the degradation of AID-NCL, cells were treated with 5-phenyl-indole-3-acetic acid (phenyl-auxin, 5-Ph-IAA) (Tocris, #7392). To block the interaction between APC/C and cdc20, cells were treated with 75 µM APCin (2-(2-Methyl-5-nitroimidazol-1-yl)ethyl N-[2,2,2-trichloro-1-(pyrimidin-2-ylamino)ethyl]carbamate, 3-(2-Methyl-5-nitro-imidazol-1-yl)-N-(2,2,2-trichloro-1-phenylamino-ethyl)-propionamide) (Sigma-Aldrich SML1503). Drugs were diluted in culture media at treatment.

### Incucyte analyses

For live cell imaging experiments, 2,000–3,000 cells were plated in 96-well plates and time-lapse analyses were performed using IncuCyte S3 Live-Cell Analysis System (Essen BioScience). Images were acquired in a single plane of focus with a 10X objective using bright-field, GFP, and RFP channels, at the indicated timepoints. Each experiment was performed in at least 3 biological replicates. Of each replicate, at least 4 technical replicates were acquired. Quantification of cell confluence and fluorescence integrated intensity was generated by the IncuCyte software. Incucyte raw data were exported as averages of the technical replicates and imported into GraphPad Prism software for statistical analyses.

### Statistical analyses

The number of replicates, and the statistical tests that were used for each experiment are specified in the relative figure legend. For statistical analysis, GraphPad Prism software was used. Data was considered statistically significant for *p* < 0.05.

## Supplementary Information

Supplementary Material 1.

## Data Availability

RNA-sequencing and TMT proteomics data are pending deposition into public data repository. Source data files are available in the Supplementary Data or from the Principal Investigator upon request.

## Abbreviations

5-Ph-IAA: 5-Phenyl-Indole-3-Acetic Acid (synthetic auxin derivative)
AAVS1: Adeno-Associated Virus Integration Site 1 (safe-harbor locus)
AID: Auxin Inducible Degron
APC/C: Anaphase Promoting Complex/Cyclosome
APCin: APC/C–Cdc20 interaction inhibitor
ATCC: American Type Culture Collection
BC: Breast Cancer
CNVs: Copy Number Variations
CPTAC: Clinical Proteomic Tumor Analysis Consortium
CRISPR/Cas9: Clustered Regularly Interspaced Short Palindromic Repeats / CRISPR-associated protein 9
Cdc20: Cell Division Cycle 20
DepMap: Cancer Dependency Map
FACS: Fluorescence-Activated Cell Sorting
FDR: False Discovery Rate
FUSCC: Fudan University Shanghai Cancer Center
GFP: Green Fluorescent Protein
GO: Gene Ontology
GSEA: Gene Set Enrichment Analysis
H1.0: Histone H1.0
HDR: Homology Directed Repair
IRES: Internal Ribosome Entry Site
LogFC: Log Fold Change
NCL: Nucleolin
NES: Normalized Enrichment Score
OsTIR1: Oryza sativa Transport Inhibitor Response 1
RFP: Red Fluorescent Protein
RNAi: RNA interference
STR: Short Tandem Repeat
TCGA: The Cancer Genome Atlas
TNBC: Triple Negative Breast Cancer
WB: Western Blot
mAID2: Mini Auxin Inducible Degron, second generation
mCherry2: Fluorescent red protein variant of mCherry
mEmerald: Green fluorescent protein variant
mMaroon1: Far-red fluorescent protein
rRNA: Ribosomal RNA
shRNA: Short hairpin RNA
siRNA: Small interfering RNA

## References

[1] Lafontaine DLJ, Riback JA, Bascetin R, Brangwynne CP. The nucleolus as a multiphase liquid condensate. Nat Rev Mol Cell Biol. 2021;22:165–82. 32873929 10.1038/s41580-020-0272-6

[2] Stenström L, et al. Mapping the nucleolar proteome reveals a spatiotemporal organization related to intrinsic protein disorder. Mol Syst Biol. 2020;16:e9469. 32744794 10.15252/msb.20209469 PMC7397901

[3] Sakthivel D, Brown-Suedel A, Bouchier-Hayes L. The role of the nucleolus in regulating the cell cycle and the DNA damage response. Adv Protein Chem Struct Biol. 2023;135:203–41. 37061332 10.1016/bs.apcsb.2023.01.001

[4] Lafita-Navarro MC, Conacci-Sorrell M. Nucleolar stress: from development to cancer. Semin Cell Dev Biol. 2023;136:64–74. 35410715 10.1016/j.semcdb.2022.04.001 PMC9883801

[5] Tiku V, Antebi A. Nucleolar function in lifespan regulation. Trends Cell Biol. 2018;28:662–72. 29779866 10.1016/j.tcb.2018.03.007

[6] Bursać S, Prodan Y, Pullen N, Bartek J, Volarević S. Dysregulated ribosome biogenesis reveals therapeutic liabilities in cancer. Trends Cancer. 2021;7:57–76. 32948502 10.1016/j.trecan.2020.08.003

[7] Corman A, Sirozh O, Lafarga V, Fernandez-Capetillo O. Targeting the nucleolus as a therapeutic strategy in human disease. Trends Biochem Sci. 2023;48:274–87. 36229381 10.1016/j.tibs.2022.09.006

[8] Orgebin E, et al. Ribosomopathies: new therapeutic perspectives. Cells. 2020;9:2080. 32932838 10.3390/cells9092080 PMC7564184

[9] Derenzini M, Montanaro L, Treré D. What the nucleolus says to a tumour pathologist. Histopathology. 2009;54:753–62. 19178588 10.1111/j.1365-2559.2008.03168.x

[10] Weeks SE, Metge BJ, Samant RS. The nucleolus: a central response hub for the stressors that drive cancer progression. Cell Mol Life Sci. 2019;76(22):4511–24. 31338556 10.1007/s00018-019-03231-0 PMC6841648

[11] Tonello F, Massimino ML, Peggion C. Nucleolin: a cell portal for viruses, bacteria, and toxins. Cell Mol Life Sci. 2022;79:271. 35503380 10.1007/s00018-022-04300-7 PMC9064852

[12] Mongelard F, Bouvet P. Nucleolin: a multiFACeTed protein. Trends Cell Biol. 2007;17:80–6. 17157503 10.1016/j.tcb.2006.11.010

[13] Berger CM, Gaume X, Bouvet P. The roles of nucleolin subcellular localization in cancer. Biochimie. 2015;113:78–85. 25866190 10.1016/j.biochi.2015.03.023

[14] Romano S, Fonseca N, Simões S, Gonçalves J, Moreira JN. Nucleolin-based targeting strategies for cancer therapy: from targeted drug delivery to cytotoxic ligands. Drug Discov Today. 2019;24:1985–2001. 31271738 10.1016/j.drudis.2019.06.018

[15] Fonseca NA, et al. GMP-grade nanoparticle targeted to nucleolin downregulates tumor molecular signature, blocking growth and invasion, at low systemic exposure. Nano Today. 2021;37:101095.

[16] Koutsioumpa M, Papadimitriou E. Cell surface nucleolin as a target for anti-cancer therapies. Recent Pat Anticancer Drug Discov. 2014;9:137–52. 24251811 10.2174/1574892808666131119095953

[17] Roger B, Moisand A, Amalric F, Bouvet P. Repression of RNA polymerase I transcription by nucleolin is independent of the RNA sequence that is transcribed. J Biol Chem. 2002;277:10209–19. 11773064 10.1074/jbc.M106412200

[18] Ugrinova I, et al. Inactivation of nucleolin leads to nucleolar disruption, cell cycle arrest and defects in centrosome duplication. BMC Mol Biol. 2007;8:1–16. 17692122 10.1186/1471-2199-8-66 PMC1976620

[19] Hua L, Yan D, Wan C, Hu B. Nucleolus and nucleolar stress: from cell fate decision to disease development. Cells. 2022;11:3017. 36230979 10.3390/cells11193017 PMC9563748

[20] Natsume T, Kiyomitsu T, Saga Y, Kanemaki MT. Rapid protein depletion in human cells by Auxin-inducible degron tagging with short homology donors. Cell Rep. 2016;15:210–8. 27052166 10.1016/j.celrep.2016.03.001

[21] Yesbolatova A, et al. The auxin-inducible degron 2 technology provides sharp degradation control in yeast, mammalian cells, and mice. Nat Commun. 2020;11:1–13. 33177522 10.1038/s41467-020-19532-z PMC7659001

[22] Fonseca NA, et al. Nucleolin overexpression in breast cancer cell sub-populations with different stem-like phenotype enables targeted intracellular delivery of synergistic drug combination. Biomaterials. 2015;69:76–88. 26283155 10.1016/j.biomaterials.2015.08.007

[23] Hanauer D, Rhodes D, Sinha-Kumar C, Chinnaiyan A. Bioinformatics approaches in the study of cancer. Curr Mol Med. 2007;7:133–41. 17311538 10.2174/156652407779940431

[24] Koboldt DC, et al. Comprehensive molecular portraits of human breast tumours. Nature. 2012;490(7418):61–70. 23000897 10.1038/nature11412 PMC3465532

[25] Tao F. 1st NCI annual meeting on clinical proteomic technologies for cancer. Expert Rev Proteomics. 2008;5:17–20. 18282119 10.1586/14789450.5.1.17

[26] Jiang YZ, et al. Genomic and transcriptomic landscape of triple-negative breast cancers: subtypes and treatment strategies. Cancer Cell. 2019;35:428–440.e5. 30853353 10.1016/j.ccell.2019.02.001

[27] Tsherniak A, et al. Defining a cancer dependency map. Cell. 2017;170:564–576.e16. 28753430 10.1016/j.cell.2017.06.010 PMC5667678

[28] Pacini C, et al. Integrated cross-study datasets of genetic dependencies in cancer. Nat Commun. 2021;12(1):1–14. 33712601 10.1038/s41467-021-21898-7 PMC7955067

[29] Cho NH, Cheveralls KC, Brunner A-D, Kim K, Michaelis AC, Raghavan P, et al. OpenCell: Endogenous tagging for the cartography of human cellular organization. Science. 2022;375(6585):eabi6983. 10.1126/science.abi6983. 10.1126/science.abi6983 PMC9119736 35271311

[30] Shen Y, Chen Y, Wu J, Shaner NC, Campbell RE. Engineering of mCherry variants with long Stokes shift, red-shifted fluorescence, and low cytotoxicity. PLoS One. 2017;12:e0171257. 28241009 10.1371/journal.pone.0171257 PMC5328254

[31] Los GV, Wood K. The HaloTag^TM^: a novel technology for cell imaging and protein analysis. In high content screening. New Jersey: Humana Press. pp.195–208. 10.1385/1-59745-217-3:195.

[32] Oceguera-Yanez F, et al. Engineering the AAVS1 locus for consistent and scalable transgene expression in human iPSCs and their differentiated derivatives. Methods. 2016;101:43–55. 26707206 10.1016/j.ymeth.2015.12.012

[33] Capece M, et al. A novel auxin-inducible degron system for rapid, cell cycle-specific targeted proteolysis. Cell Death Differ. 2023;30:2078. 10.1038/s41418-023-01191-4. 37537305 10.1038/s41418-023-01191-4 PMC10482871

[34] Abdelmohsen K, Gorospe M. RNA Biology RNA-binding protein nucleolin in disease. RNA Biol. 2012;799:799–808. 10.4161/rna.19718 PMC3495746 22617883

[35] Bajar BT, et al. Fluorescent indicators for simultaneous reporting of all four cell cycle phases. Nat Methods. 2016;13:993–996. Preprint at 10.1038/nmeth.4045. 10.1038/nmeth.4045 PMC5548384 27798610

[36] EbastienStorck S, Thiry M, Bouvet P. Conditional knockout of nucleolin in DT40 cells reveals the functional redundancy of its RNA-binding domains. Biol Cell. 2009;101:153–71. 18637790 10.1042/BC20080054

[37] Sawicka A, Seiser C. Histone H3 phosphorylation – a versatile chromatin modification for different occasions. Biochimie. 2012;94:2193. 22564826 10.1016/j.biochi.2012.04.018 PMC3480636

[38] Lens SMA, Medema RH. Cytokinesis defects and cancer. Nat Rev Cancer. 2018;19(1):32–45. 10.1038/s41568-018-0084-6 30523339

[39] Steigemann P, et al. Aurora B-mediated abscission checkpoint protects against tetraploidization. Cell. 2009;136:473–84. 19203582 10.1016/j.cell.2008.12.020

[40] De Lange J, et al. Defective sister chromatid cohesion is synthetically lethal with impaired APC/C function. Nat Commun. 2015;6(6):8399. 26423134 10.1038/ncomms9399 PMC4600715

[41] Grinstein E, et al. Cell cycle-controlled interaction of nucleolin with the retinoblastoma protein and cancerous cell transformation. J Biol Chem. 2006;281:22223–35. 16698799 10.1074/jbc.M513335200

[42] Hoffmann I. Centrosomes in mitotic spindle assembly and orientation. Curr Opin Struct Biol. 2021;66:193–8. 10.1016/j.sbi.2020.11.003 33296732

