# Supplementary material for: Acute degradation of nucleolin reveals its novel functions in cell cycle progression and cell division in triple negative breast cancer
Source: J Exp Clin Cancer Res. 2025 Jul 14;44:204. doi: 10.1186/s13046-025-03401-y (PMC12257848; doi:10.1186/s13046-025-03401-y)
Supplement: Supplementary file 1 — Supplementary Material 1. [file 13046_2025_3401_MOESM1_ESM.zip › Mills et al Supp Info Rev1.docx]

**Acute degradation of Nucleolin reveals its novel functions in cell cycle progression and cell division in Triple Negative Breast Cancer**

Joseph Mills, Anna Tessari, Vollter Anastas, Damu Sunilkumar, Nastaran Samadi Rad, Saranya Lamba, Ilaria Cosentini, Ashley Reers, Zirui Zhu, Wayne O. Miles, Vincenzo Coppola, Emanuele Cocucci, Thomas J. Magliery, Heather Shive, Alexander E. Davies, Lara Rizzotto, Carlo M. Croce, and Dario Palmieri

**Supplementary Data:**

- **Supplementary Methods**

**Plasmids**

All the newly generated plasmids reported in this study were created by Gibson Assembly using NEBuilder HiFi DNA Assembly Master Mix (New England Biolabs, E2621L) as per manufacturer’s instructions. The ROLECCS-V2-AS plasmid was previously described^1^. The OsTIR1(F74G)-IRES-H1-mMaroon was generated by replacing the puromycin cassette of Addgene plasmid 140536 (^2^) with the IRES-H1-mMaroon cassette of Addgene plasmid 83842 (pLL3.7m-mTurquoise2-SLBP(18-126)-IRES-H1-mMaroon1)^3^.

To generate the donor plasmids for endogenous *NCL* editing, the genomic region (~2000 bp) surrounding the natural start codon on exon 1 was cloned into the pUC19 vector (New England Biolabs, N3041S) by Gibson Assembly. Genomic DNA from MDA-MB-231 cells was used as a template to amplify the *NCL* genomic region (Chromosome 2: 231,453,531-231,464,484, Transcript: NCL-201 ENST00000322723.9) of 1 kb upstream and 1 kb downstream the NCL translation start codon. These regions were further used as homology arms for HDR-mediated CRISPR/Cas9-mediated knock-ins. The mAID-mCherry cassette was amplified from Addgene plasmid 72830 (pMK292)^4^ and mAID-HaloTag was amplified from Addgene plasmid 112852^5^. Schematic overview of the tagging vectors is reported in Figure 2A.

To construct the CRISPR/Cas9 NCL-5’ gene targeting vector, a single guide RNA (sgRNA) (5’- CTTCGCGAGCTTCACCATGA-3’) was designed (http://crispr.mit.edu) to target NCL translation start site and the targeting sequence was cloned into pX330-U6-Chimeric_BB-CBh-hSpCas9-hGem (1/110) (Addgene#71707)^6^ according to standard protocols^7^. The same protocol was followed to clone the sgRNA used for *OsTIR1* insertion in the *AAVS1* locus^4,8^.

All the plasmids generated in the study will be available on Addgene (www.addgene.org).

**Protein Extraction, Western Blots**

For total protein extractions, cells were collected and washed with PBS before adding adequate amount of lysis buffer (1% NP-40, 1 mM EDTA pH 8.00, 50 mM Tris-HCl pH 7.5, 150 mM NaCl) containing a protease and phosphatase inhibitor cocktail (cOmplete^TM^, EDTA-free Protease Inhibitor Cocktail, Millipore Sigma, #COEDTAF-RO; PhosSTOPTM inhibitor tablets, Millipore Sigma, #PHOSS-RO). Protein concentration was estimated by Bradford assay (Biorad, #5000006). After denaturation at 100 ^o^C for 10 minutes, equal amounts of protein (10-30 μg) were separated using SDS-PAGE, loading samples on 4-20% Mini-PROTEAN® TGX™ Precast Protein Gels (Biorad, #456109). Proteins were then transferred to a 0.45 μm nitrocellulose membrane (Biorad, #1620094) and blocked in 5% non-fat milk or bovine serum albumin in TBST for 1 hour at room temperature. Following blocking, membranes were probed overnight with primary antibodies at 4 ^o^C. The next day, membranes were washed three times with TBST before incubation with secondary antibody at room temperature for 1hour. Detection was performed using Pierce™ ECL Western Blotting Substrate (ThermoFisher Scientific, #32209) and either X-ray blue films, in dark room, or the Li-COR Odyssey FC Imager (Li-Cor Bioscience). When necessary, membranes were stripped using Western BLoT Stripping Buffer (Takara Bio, #T7135A) and re-probed with the desired antibody.

Primary antibodies used were anti-NCL (D4C7O) Rabbit mAb (Cell Signaling Technology, #14574); anti-GFP (B-2) Mouse mAb (Santa Cruz Biotechnology, sc-9996); anti-OsTIR1 Rabbit polyclonal Antibody (MBL Bio, #PD048); anti-GAPDH (14C10) Rabbit mAb (HRP Conjugate (Cell Signaling Technology, #12231). Secondary antibodies used were WesternSure® Goat anti-Rabbit HRP Secondary Antibody (Li-Cor Bioscience, # 926-80011), WesternSure® Goat anti-Mouse HRP Secondary Antibody (Li-Cor Bioscience, 926-80010), HRP-conjugated anti-mouse IgG (Millipore Sigma, #NA931V), HRP-conjugated anti-rabbit IgG (Millipore Sigma, #NA934V).

**RNA sequencing**

For RNA sequencing experiment, two independent MDA-MB-231 AID-NCL/OsTIR1 clones were grown in a T175cm flask and either not treated or treated with 2 μM 5-Ph-IAA for 16 hours. Cells were then collected, and RNA was extracted using TRIzol RNA Isolation Reagent (ThermoFisher, #15596026). Library preparation and Next Generation Sequencing were performed at UCLA Technology Center for Genomics and Bioinformatics (TCGB). The library type was Illumina-RNA (rRNA depleted) and Next-Generation Sequencing was performed using Illumina NovaSeq-S1-PE 150 Cycle, with read lengths of 2x150-bp (paired ends). After trimming the library adapter sequences from the raw reads using TrimGalore (github.com/FelixKrueger/TrimGalore), hisat2 (github.com/DaehwanKimLab/hisat2) was used to map the reads to the reference human genome (GRCh38). The output SAM files were converted to BAM files, sorted by index. Reads quality across samples was tested using MultiQC^9^. Gencode gtf file (version 39) was used for gene annotation and counts generation using feature-counts^10^. Raw counts were filtered for low expressed genes following the library size normalization (TMM- Trimmed Mean of M values) using edgeR^11^. The identification of differentially expressed genes between cases and controls was conducted using the R package limma^12^. Adjusted p-values were calculated using the Benjamini and Hochberg method. Heatmaps were generated using the R package pheatmap (https://github.com/raivokolde/pheatmap).

**Data analysis of TCGA, FUSCC, and CPTAC**

For TCGA analysis, data was downloaded from The Cancer Genome Atlas (TCGA) database using the Xena browser^13^, provided by University of California Santa Cruz (UCSC). Data from the TCGA Breast Cancer (BRCA) was selected, and samples with PAM50 phenotype designations^14^ were identified. RNA expression data of NCL was obtained for each patient sample. Data points were downloaded, and GraphPad was used to compare subgroups of NCL gene expression and the PAM50 subgroups in order to visualize NCL gene expression levels in the 5 different phenotypes: normal-like, luminal A, luminal B, HER2-enriched, and basal-like. Statistical significance was calculated using Kruskal-Wallis one-way ANOVA with multiple comparisons.

Analysis of NCL expression in different stages of tumor development were analyzed using the same database. Patient samples were categorized based on pathological stage and NCL expression data was obtained for each patient sample. Data was plotted for stages I-IV. Statistical significance was calculated Kruskal-Wallis one-way ANOVA with multiple comparisons.

Similarly, RNAseq FPKM (Fragments Per Kilobase per Million mapped fragments) count data from the FUSCC TNBC cohort were obtained from node OEZ000398 on [www.biosino.org](http://www.biosino.org). RNAseq Data for NCL were categorized as basal or non-basal and statistical significance was calculated via two-way Mann-Whitney test.

Protein abundance data for breast cancer patient samples from CPTAC was obtained from cBioportal^15–17^. Protein samples were categorized into PAM50 subgroups and all non-basal tumors were grouped together. Statistical significance was calculated via one-way ANOVA or Mann-Whitney test.

**GSEA**

Gene Set Enrichment Analysis (GSEA) was performed on the RNAseq based on normalized count files obtained from the RNAseq comparing samples which were treated and not treated with 2 μM 5-Ph-IAA for 16 hours. Enrichment analysis was performed using the Hallmark Gene Set available from the GSEA database. 1000 permutations were run for each enrichment analysis and statistics were calculated within the GSEA software based on the probability of the obtained calculated enrichment score as compared to the generated permutations. Statistical testing was adjusted for multiple hypothesis testing. For pre-ranked GSEA analysis utilizing patient data, Spearman correlations rho-values between NCL RNA or protein levels and other factors RNA or protein levels were calculated on cBioPortal^15–17^. Genes were ranked from most positively correlated with NCL to most negatively correlated with NCL and this ranked list served as the input to the GSEA. Enrichment analysis was performed using the Gene Ontology Biological Processes Gene Set available from the GSEA database. Enrichment analysis was performed using 1000 permutations as stated above, comparing the generated permutations enrichment scores to that of the input pre-ranked list. Statistical analysis was performed within the GSEA software and statistical testing was adjusted for multiple hypothesis testing.

**Flow Cytometry analyses**

MDA-MB-231 AID-NCL/OsTIR1/H1.0-mMaroon1 cells were collected, washed in 1X PBS before being fixed in ice-cold 70% ethanol, and stored at -20 ^o^C overnight. Cells were then pelleted and washed in 1X PBS and incubated with Phospho-Histone H3 (Ser10) (D2C8) XP Rabbit mAb (Cell Signaling Technology, #3377) in 1% BSA/PBS for 90 minutes at room temperature (RT). After primary antibody staining, cells were washed with 1x PBS, and incubated with Goat anti-rabbit Alexa 488 secondary antibody (Life Technologies, #A-11008) in 1% BSA/PBS. Finally, cells were pelleted, washed with 1X PBS, and stained with propidium iodide staining solution (1X PBS containing 10 mg/mL propidium iodide (Thermo Fisher Scientific, #P1304MP), 0.05% Triton X-100 (Millipore Sigma, #[9036-19-5](https://www.sigmaaldrich.com/US/en/search/9036-19-5?focus=products&page=1&perpage=30&sort=relevance&term=9036-19-5&type=cas_number)), 2.5 µg/mL RNAse A (Thermo Fisher Scientific, # EN0531)). Flow cytometry analysis was performed using LSRFortessa (Beckman-Dickinson) Flow Cytometer, modeling at least 5,000 events per sample. Gating was designed to quantitate the absolute population of cells with a 4N content of DNA, and either positively (4N; pH3+) or negatively (4N; pH3-) stained for pH3(S10) antibody, using the FloJo software. Cell cycle analyses (based on PI staining alone) were performed with ModFit software, v5.0. Two independent clones were analyzed, for a total of three experiments. Each experiment was performed in technical duplicate, and data were normalized for the untreated controls. Significance was calculated using GraphPad Prism software. Welch’s t-test was applied.

**Immunofluorescence**

The formation of bi-nucleated cells was evaluated by immunofluorescence as follows. Cells were plated on sterile glass coverslips (Gold Seal Cover Glass, thickness 1.5, Thermo Fisher, #3406) pretreated with poly-L-ornithine solution (Sigma Aldrich, #P4957) in 6-well plates in regular medium. The next day, cells were treated with 2 µM 5-Ph-IAA for 72 hours or left untreated. Following treatment, medium was aspirated, and coverslips were washed with 1X PBS and fixed with cold 4% PFA/PBS pH 7.4 (Paraformaldehyde Solution, 4% in PBS, Thermo Scientific™, #J19943.K2) for 15 minutes at RT. Fixative solution was then removed, and cells were incubated with 0.3 M Glycine solution (Glycine USP, Gojiara Fine Chemicals, #GC1004, dissolved in dH_2_O and filtered) for 5 minutes at RT. Coverslips were then rinsed with 1X PBS and incubated with 1X PBS for 5 minutes at RT. Cells were then permeabilized with 0.2% Triton X-100 in 1X PBS for 10 minutes at RT. Following a wash with 1X PBS, cells were blocked in 5% BSA/PBS (filtered) for 1 hour at RT. Next, coverslips were incubated with anti-Lamin A/C (4C11) Mouse mAb (Cell Signaling Technology, #4777), diluted in 1% BSA/PBS filtered solution, for 1 hour at RT in a covered humidified chamber. Coverslips were then rinsed with 1X PBS and washed two times for 10 minutes each with 1X PBS. Next, cells were incubated with Goat anti-Mouse IgG Antibody AlexaFluor™ 488 (Fisher Scientific, #A11029) and Phalloidin-iFluor 647 (Abcam, #ab176759) in 1% BSA/PBS filtered solution for 1 hour at RT in a covered humidified chamber. Coverslips were then rinsed with 1X PBS and washed three times for 10 minutes each with 1X PBS. Coverslips were mounted on Superfrost Plus Microscope Slides (Fisherbrand, #12-550-15), using VECTASHIELD Antifade Mounting Medium with DAPI (Vector Laboratories, #H-1200-10). Slides were left at RT overnight, before evaluation. Images were captured in a single plane of focus at 60x magnification using an EVOS™ M5000 Imaging System (EVOS™ M5000 Software). Bi-nucleated cells were defined as single cells identifiable by an actin border positive to phalloidin staining (magenta), with two separate nuclei encased by nuclear envelope positive to Lamin A/C staining (green). For quantification purposes, at least 15 independent fields were captured, for a total of at least 100 cells for each sample. The percentage of bi-nucleated cells per image was used to generate the comparison between treated and not treated cells. Data were analyzed using GraphPad Prism software. Statistical significance was calculated using one-way ANOVA with Sidak’s multiple comparison test.

**TMT quantitative proteomic analyses.**

Quantitative proteomics was performed as previously described^18,19^. Briefly, cells were plated in T175cm flasks and grown to subconfluency. Cells were either subjected to 2 µM 5-Ph-IAA for 24 hours or remained untreated. Cell lysis was performed using 8M Urea, 50 mM Triethylamonium bicarbonate (TEAB) buffer containing protease and phosphatase inhibitors (Roche), as described in “Protein Extraction”. The samples were then sonicated for 30 seconds on ice (amplitude 25%), and concentration was estimated Pierce^TM^ BCA Protein Assay Kit (Thermo Fisher Scientific, Cat. 23225). Tryptic digestion, Tandem Mass Tag (TMT) labelling, fractionation, mass spectrometry, and validation analyses were performed by Bioinformatics Solutions Inc., using at least 100 μg of extracted proteins. Peptide spectra were searched using the Uniport validated human proteome and Peaks Studio (v11), using a 15-ppm precursor ion tolerance for peptide mapping. A decoy fusion method was employed to determine if peptide-spectrum matchings are true matchings or likely false positives. A cut-off of 1% false-discovery-rate was used to filter poor peptide matchings. Relative abundance (Log2 Fold Change) and significance (-10Log10 of *p*-value) were calculated by comparing the peptide quantification values control and 5-Ph-IAA treated samples, via Peaks Studio. Data were considered significant for -10Log10 of *p*-value >13.

**Live-cell imaging**

Cells were seeded at a density of 1,000 cells per well in PhenoPlate™ 384-well microplates and incubated overnight at 37°C in a CO₂ incubator to allow for adherence. The following day, cells were either left untreated or treated with 2 µM 5-Ph-IAA.To monitor cell division, including cytokinesis and multinucleation events, time-lapse imaging was performed using the Opera Phenix High-Content Screening System (Revvity). Imaging was conducted at 20X magnification in both the brightfield and infrared (mMaroon) fluorescence channels to capture cellular morphology and nuclear structures. Live-cell imaging was carried out every 3 minutes over a 16-hour period at each time point of interest to track the progression of cell division. Automated image acquisition parameters were optimized to maximize the signal-to-noise ratio while minimizing phototoxicity. Image analysis was performed using Harmony™ software. Videos of identified multinucleated cells in the 5-Ph-IAA treatment group were exported at a playback speed of 3 frames per second.

- **Supplementary Figure Legends**

**Figure S1. Increased NCL protein levels in Basal Like breast tumors.**

**A)** NCL protein levels among different breast tumor subtypes compared to the normal-like breast cancer samples. Protein levels were obtained from 122 patient samples (Normal-like, n=5; Luminal A, n=57; Luminal B, n=17; HER2-enriched, n=14; Basal Like, n=29 available through the Clinical Proteomic Tumor Analysis Consortium (CPTAC). **B)** Comparison of NCL protein levels in Basal Like (n=29) vs pooled non-Basal Like (n=93) BC, as shown in A. Significance was defined using Mann-Whitney test. **: *p* < 0.01.

**Figure S2. Development of an Auxin Inducible Degron for NCL.**

**A-B)** Cancer DepMap data from RNAi (**A**) and CRISPR-KO (**B**) screen databases (as indicated) show NCL Perturbation Score across Breast Cancer cell lines (empty dots) or total cell lines (black dots). This score measures the effect of knocking out a gene on cell viability and is derived from large-scale CRISPR or RNAi screens across numerous cancer cell lines. DepMap defines genes as commonly essential when their perturbation score is in the top 10% of all genes, and a dependency score of -0.5 or lower is considered a significant dependency. For breast cancer cell lines, the highest observed score was -0.52 (MDA-MB-361 cells, an ER-positive/PgR-negative luminal mammary carcinoma cell line) **C)** Copy number variation (CNV) analysis of genes in MDA-MB-231 cells from the Cancer DepMap data. Blue dots represent individual genes, aligned based on their absolute copy number. Nucleolin (*NCL*) is highlighted in red. **D)** Schematic representation of the plasmid used as a donor for *AAVS1* site-specific integration of the *OsTIR1*(F74G)-mEmerald fusion gene (used for the generation of clones C10.C4 and C10.D9). **E**) Schematic representation of the plasmid used as a donor for *AAVS1* site-specific integration of the vector co-expressing *OsTIR1*(F74G) and histone H1-mMaroon1 fusion protein (used for the generation of clones C10.C1 and C10.C2). IRES: internal ribosome entry site. **F-G)** Western blot analyses for effective integration (F) and biological activity (G) of OsTIR1(F74G) in MDA-MB-231 AID-NCL homozygous clones (see also Figure 2 and S2E) using the indicated antibodies. GAPDH was used as loading control. For degradation experiments, clones were treated for 24 h with 5-Ph-IAA. **H)** Representative images of Incucyte experiments on MDA-MB-231 mCherry2/Halo-AID-NCL/OsTIR1-mEmerald cells (see also Figure 2G). The RFP channel was used to detect mCherry2-AID-NCL, and the GFP channel was used to assess OsTIR1-mEmerald expression and localization. Cells were left untreated or treated with for 2 h with 5-Ph-IAA (see also Figure 2G). **I)** Quantitative analysis of mCherry/NCL intensity from Incucyte experiments on the indicated cell clones, not treated, or treated with 5-Ph-IAA. RFP integrated intensity per image was normalized for t=0. The experiment was performed on 9 biological replicates, with 5 technical replicates. Error bars show standard deviation.

**Figure S3. AID-dependent NCL abrogation affects cell proliferation.**

**A-B)** Growth curves comparing parental MDA-MB-231 and all the gene-edited derivative clones, left untreated (A) or treated with 2 μM 5-Ph-IAA (B). **(C)** Growth curve of parental MDA-MB-231 cells, with or without treatment with 2 μM 5-Ph-IAA, **(D)** Growth curve of MDA-MB-231 AID-NCL clones (not expressing OsTIR1) with or without treatment with 2 μM 5-Ph-IAA. **(E-F)** Growth curve of two AID-mCherry2-NCL OsTIR1 MDA-MB-231 clones, with or without treatment with 2 μM 5-Ph-IAA for up to 140 hours. All the growth curves in this experiment and in main Figure 3 are summarized in Figure S3A-B, and individual comparisons are shown in independent panels. Cell proliferation was monitored with Incucyte live-cell imaging system. All the growth curves were performed in 5 biological replicates with 5 technical replicates. Error bars show standard deviation. Statistical significance was calculated using 2-way ANOVA with Tukey’s multiple comparison test. Where significant, the exact *p*-value is reported (E-F).

**Figure S4. NCL acute abrogation induces defects of cell cycle progression (relative to Figure 4B).** Quantitative analyses of the relative percentage of cells in G1 (A) and S (B) phase of the cell cycle, as shown in Figure 4B. Data are representative of two independent clones analyzed in three experiments performed in technical duplicate and normalized for the percentage of cells in G1 or S in untreated controls. Significance was calculated using Welch’s t-test.

**Figure S5. NCL expression correlates with genes involved in RNA metabolism and chromosomal dynamics in BC patients, and its abrogation enhances sensitivity to APC inhibitors.**

**A)** Enrichment plots of eight among the top ten most enriched GO terms containing genes positively correlating with NCL RNA expression in BC patients (see also Figure 1A and Figure 5A). **B)** Volcano plot of differentially abundant proteins upon NCL abrogation in MDA-MB-231 OsTIR1/AID-NCL cells (clone C10.C4), detected by TMT-proteomic analysis. Reported data is the full range of detected proteins as in Figure 5, with a maximum -10log_10_(p-value) of 200. **C)** Incucyte growth curves, referred to end-point experiments shown in Figure 5D-E, of two independent MDA-MB-231 OsTIR/AID-NCL clones, left untreated or treated with indicated doses 5-Ph-IAA, APCin, or combination, for up to 72 h. Incucyte live-cell imaging system was used to monitor cell proliferation. Cell confluence was normalized for t=36’. The experiment was performed in three biological replicates, with five technical replicates. Error bars show standard deviation.

- **Supplementary Tables**

**Table S1: Deregulated proteins upon NCL acute degradation (GO Term: Ribosome Biogenesis)**

| **Protein Name** | **FC (Log2)** | **Significance -10log10(p-value)** |
| --- | --- | --- |
| RPL14 | -1.4 | 84.7 |
| RPS9 | -1.3 | 43.4 |
| RPS4X | -0.8 | 39.0 |
| RPS16 | -1.0 | 37.9 |
| RPS24 | -0.7 | 34.1 |
| RPS7 | -0.7 | 33.7 |
| RPS13 | -0.8 | 30.6 |
| RPL6 | -0.8 | 30.3 |
| RPL7A | -0.7 | 28.6 |
| RPL10A | -0.6 | 28.1 |
| RPS5 | -0.6 | 28.1 |
| RPS23 | -0.6 | 25.4 |
| RPL7 | -0.8 | 24.6 |
| RPL10 | -0.7 | 22.0 |
| RPS15A | -0.7 | 20.4 |
| RSL1D1 | -0.6 | 19.1 |
| RBM34 | -0.8 | 17.9 |
| EXOSC3 | 0.6 | 17.8 |
| RPS11 | -0.5 | 17.8 |
| SERBP1 | 0.5 | 17.7 |
| RPS6 | -0.6 | 17.6 |
| RPS17 | -0.5 | 16.4 |
| RPS8 | -0.5 | 16.3 |
| RPL35 | -0.7 | 15.1 |
| RPL11 | -0.5 | 14.9 |
| DDX52 | -0.8 | 14.9 |
| UTP3 | 0.8 | 14.8 |
| RPL5 | -0.4 | 14.3 |
| UTP14A | -0.7 | 14.2 |
| NAT10 | -0.4 | 13.8 |
| EXOSC8 | 0.5 | 13.8 |

**Table S2: Deregulated proteins upon NCL acute degradation (GO Term: Chromosome Segregation)**

| **Gene** | **FC (Log2)** | **Significance -10log10(p-value)** |
| --- | --- | --- |
| SLC25A5 | -1.6 | 45.5 |
| KIF2A | -1.4 | 26.3 |
| MAP1S | -1.2 | 19.6 |
| SMARCD1 | -0.9 | 18.7 |
| TPX2 | -1.2 | 18.2 |
| EML4 | -0.6 | 16.9 |
| RPS3 | -0.4 | 13.7 |

- **Extended Data**

**Extended Data 1**

Differential gene expression from RNA sequencing data performed in control vs 5-Ph-IAA treated cells, as described in Figure 3. Table includes all the identified expressed genes, independently on statistical significance, which is also reported.

**Extended Data 2**

Differential gene expression from RNA sequencing data performed in control vs 5-Ph-IAA treated cells, as described in Figure 3. Table specifically includes genes involved in the G2/M transition pathway.

**Extended Data 3**

Differential protein levels from proteomics data performed in control vs 5-Ph-IAA treated cells, as described in Figure 5 and S5. Table includes all the identified proteins, independently on statistical significance, which is also reported. See also Table S1 and S2.

**Supplementary video**

Live-cell microscopy imaging of MDA-MB-231 AID-NCL/OsTIR1/H1.0-mMaroon1 after 5-Ph-IAA treatment. Imaging started at 20 h post treatment and was carried out every 3 minutes over a 16-hour period. Brightfield and infrared fluorescence (H1.0-mMaroon1, labeling chromatin) is shown.

**Supplementary References**

1. Capece, M. *et al.* A novel auxin-inducible degron system for rapid, cell cycle-specific targeted proteolysis. *Cell Death Differ* (2023) doi:10.1038/s41418-023-01191-4.

2. Yesbolatova, A. *et al.* The auxin-inducible degron 2 technology provides sharp degradation control in yeast, mammalian cells, and mice. *Nat Commun* 11, 1–13 (2020).

3. Bajar, B. T. *et al.* Fluorescent indicators for simultaneous reporting of all four cell cycle phases. *Nature Methods* vol. 13 993–996 Preprint at https://doi.org/10.1038/nmeth.4045 (2016).

4. Natsume, T., Kiyomitsu, T., Saga, Y. & Kanemaki, M. T. Rapid Protein Depletion in Human Cells by Auxin-Inducible Degron Tagging with Short Homology Donors. *Cell Rep* 15, 210–218 (2016).

5. Gu, B., Posfai, E. & Rossant, J. Efficient generation of targeted large insertions by microinjection into two-cell-stage mouse embryos. *Nature Biotechnology 2018 36:7* 36, 632–637 (2018).

6. Gutschner, T., Haemmerle, M., Genovese, G., Draetta, G. F. & Chin, L. Post-translational Regulation of Cas9 during G1 Enhances Homology-Directed Repair. *Cell Rep* 14, 1555–1566 (2016).

7. Ran, F. A. *et al.* Genome engineering using the CRISPR-Cas9 system. *Nat Protoc* 8, 2281–2308 (2013).

8. Oceguera-Yanez, F. *et al.* Engineering the AAVS1 locus for consistent and scalable transgene expression in human iPSCs and their differentiated derivatives. *Methods* 101, 43–55 (2016).

9. Ewels, P., Magnusson, M., Lundin, S. & Käller, M. MultiQC: summarize analysis results for multiple tools and samples in a single report. *Bioinformatics* 32, 3047–3048 (2016).

10. Frankish, A. *et al.* GENCODE: reference annotation for the human and mouse genomes in 2023. *Nucleic Acids Res* 51, D942–D949 (2023).

11. Robinson, M. D., McCarthy, D. J. & Smyth, G. K. edgeR: a Bioconductor package for differential expression analysis of digital gene expression data. *Bioinformatics* 26, 139–140 (2010).

12. Ritchie, M. E. *et al.* limma powers differential expression analyses for RNA-sequencing and microarray studies. *Nucleic Acids Res* 43, e47 (2015).

13. Goldman, M. J. *et al.* Visualizing and interpreting cancer genomics data via the Xena platform. *Nature Biotechnology 2020 38:6* 38, 675–678 (2020).

14. Jiang, Y. Z. *et al.* Genomic and Transcriptomic Landscape of Triple-Negative Breast Cancers: Subtypes and Treatment Strategies. *Cancer Cell* 35, 428-440.e5 (2019).

15. Cerami, E. *et al.* The cBio cancer genomics portal: an open platform for exploring multidimensional cancer genomics data. *Cancer Discov* 2, 401–404 (2012).

16. Gao, J. *et al.* Integrative analysis of complex cancer genomics and clinical profiles using the cBioPortal. *Sci Signal* 6, (2013).

17. de Bruijn, I. *et al.* Analysis and Visualization of Longitudinal Genomic and Clinical Data from the AACR Project GENIE Biopharma Collaborative in cBioPortal. *Cancer Res* 83, 3861–3867 (2023).

18. Chavdoula, E. *et al.* Transcriptional regulation of amino acid metabolism by KDM2B, in the context of ncPRC1.1 and in concert with MYC and ATF4. *Metabolism* 150, (2024).

19. Anastas, V. *et al.* KDM2B is required for ribosome biogenesis and its depletion unequally affects mRNA translation. doi:10.1101/2024.05.22.595403.
