# Supplementary material for: Acute degradation of nucleolin reveals its novel functions in cell cycle progression and cell division in triple negative breast cancer
Source: J Exp Clin Cancer Res. 2025 Jul 14;44:204. doi: 10.1186/s13046-025-03401-y (PMC12257848; doi:10.1186/s13046-025-03401-y)
Supplement: Supplementary file 1 — Supplementary Material 1. [file 13046_2025_3401_MOESM1_ESM.zip › Supplementary Figures.pdf]

Figure S1

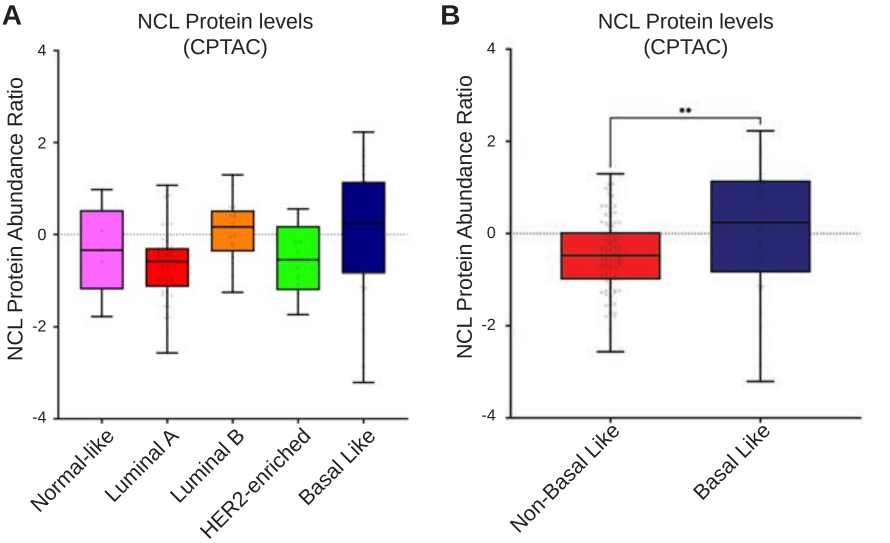

A
NCL Protein levels (CPTAC)
NCL Protein Abundance Ratio
4
2
0
-2
-4
Normal-like
Luminal A
Luminal B
HER2-enriched
Basal Like
B
NCL Protein levels (CPTAC)
NCL Protein Abundance Ratio
**
4
2
0
-2
-4
Non-Basal Like
Basal Like

Figure S2

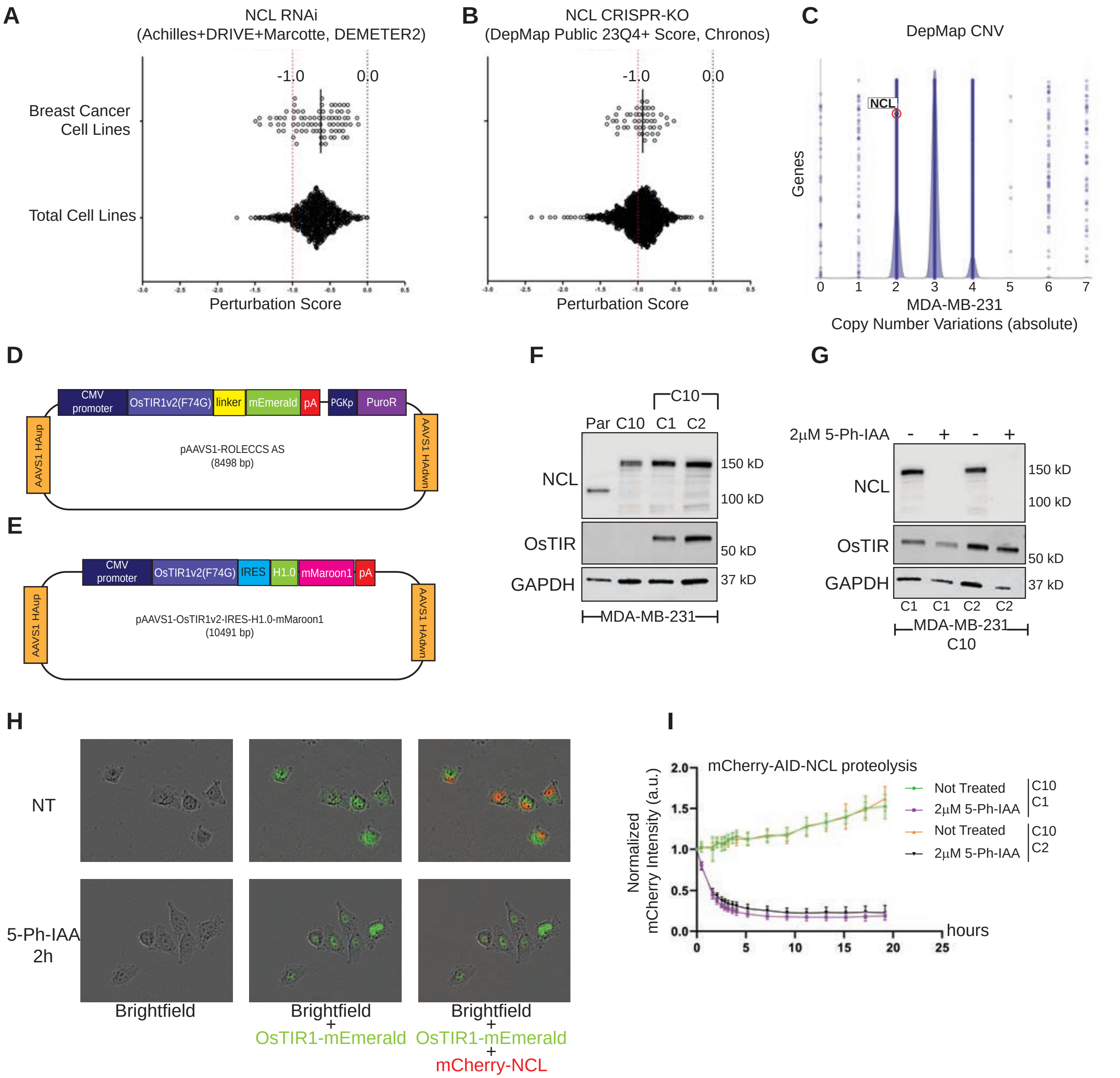
A
NCL RNAi
(Achilles+DRIVE+Marcotte, DEMETER2)
Breast Cancer Cell Lines
Total Cell Lines
-1.0
0.0
Perturbation Score
B
NCL CRISPR-KO
(DepMap Public 23Q4+ Score, Chronos)
Perturbation Score
C
DepMap CNV
Genes
NCL
0 1 2 3 4 5 6 7
MDA-MB-231
Copy Number Variations (absolute)
D
CMV promoter
OsTIR1v2(F74G)
linker
mEmerald
pA
PGKp
PuroR
AAVS1 HAup
AAVS1 HAdwn
pAAVS1-ROLECCS AS
(8498 bp)
E
CMV promoter
OsTIR1v2(F74G)
IRES
H1.0
mMaroon1
pA
AAVS1 HAup
AAVS1 HAdwn
pAAVS1-OsTIR1v2-IRES-H1.0-mMaroon1
(10491 bp)
F
C10
Par C10 C1 C2
NCL
OsTIR
GAPDH
150 kD
100 kD
50 kD
37 kD
MDA-MB-231
G
2μM 5-Ph-IAA - + - +
NCL
OsTIR
GAPDH
150 kD
100 kD
50 kD
37 kD
C1 C1 C2 C2
MDA-MB-231
C10
H
NT
5-Ph-IAA
2h
Brightfield
Brightfield
+
OsTIR1-mEmerald
Brightfield
+
OsTIR1-mEmerald
+
mCherry-NCL
I
mCherry-AID-NCL proteolysis
Normalized
mCherry Intensity (a.u.)
2.0
1.5
1.0
0.5
0.0
0 5 10 15 20 25
hours
Not Treated
2μM 5-Ph-IAA
C10
C1
Not Treated
2μM 5-Ph-IAA
C10
C2

Figure S3

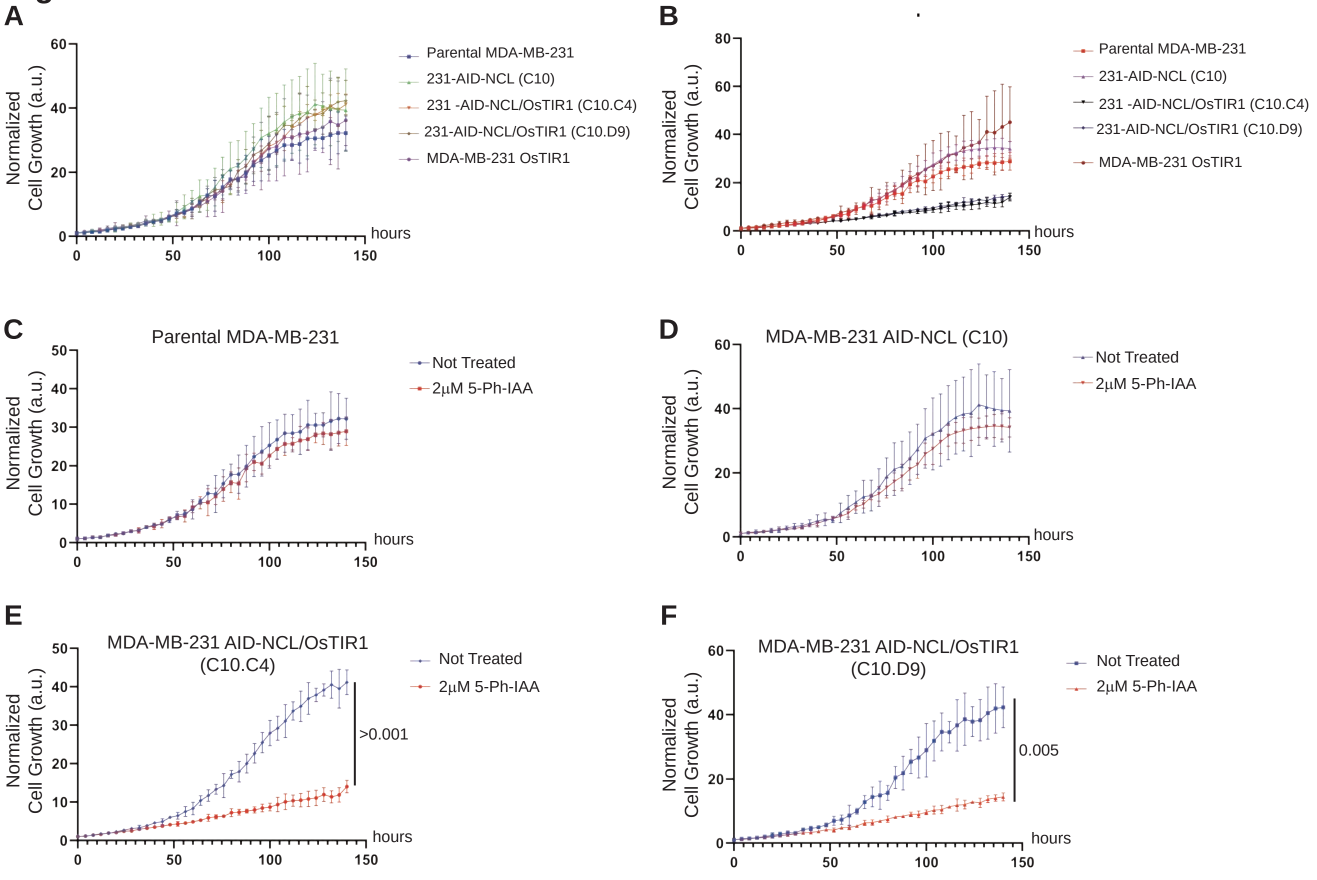
A
Normalized Cell Growth (a.u.)
0
20
40
60
0
50
100
150
hours
Parental MDA-MB-231
231-AID-NCL (C10)
231 -AID-NCL/OsTIR1 (C10.C4)
231-AID-NCL/OsTIR1 (C10.D9)
MDA-MB-231 OsTIR1
B
Normalized Cell Growth (a.u.)
0
20
40
60
80
0
50
100
150
hours
Parental MDA-MB-231
231-AID-NCL (C10)
231 -AID-NCL/OsTIR1 (C10.C4)
231-AID-NCL/OsTIR1 (C10.D9)
MDA-MB-231 OsTIR1
C
Parental MDA-MB-231
Normalized Cell Growth (a.u.)
0
10
20
30
40
50
0
50
100
150
hours
Not Treated
2µM 5-Ph-IAA
D
MDA-MB-231 AID-NCL (C10)
Normalized Cell Growth (a.u.)
0
20
40
60
0
50
100
150
hours
Not Treated
2µM 5-Ph-IAA
E
MDA-MB-231 AID-NCL/OsTIR1 (C10.C4)
Normalized Cell Growth (a.u.)
0
10
20
30
40
50
0
50
100
150
hours
Not Treated
2µM 5-Ph-IAA
>0.001
F
MDA-MB-231 AID-NCL/OsTIR1 (C10.D9)
Normalized Cell Growth (a.u.)
0
20
40
60
0
50
100
150
hours
Not Treated
2µM 5-Ph-IAA
0.005

Figure S4

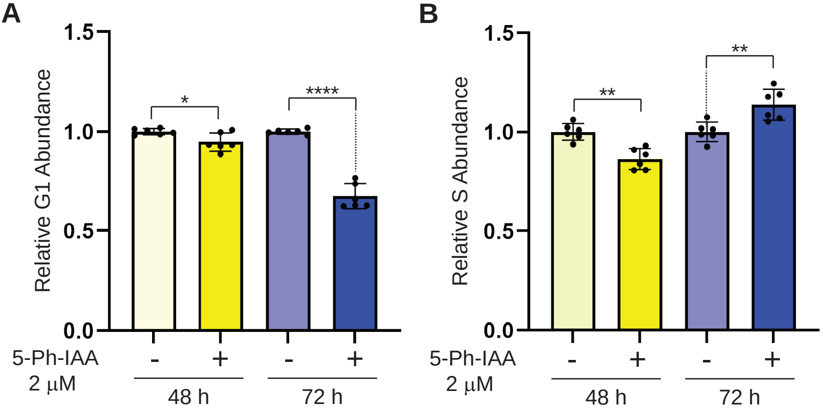

A
1.5
1.0
0.5
0.0
Relative G1 Abundance
*
****
5-Ph-IAA
2 µM
-
+
-
+
48 h
72 h
B
1.5
1.0
0.5
0.0
Relative S Abundance
**
**
5-Ph-IAA
2 µM
-
+
-
+
48 h
72 h

Figure S5

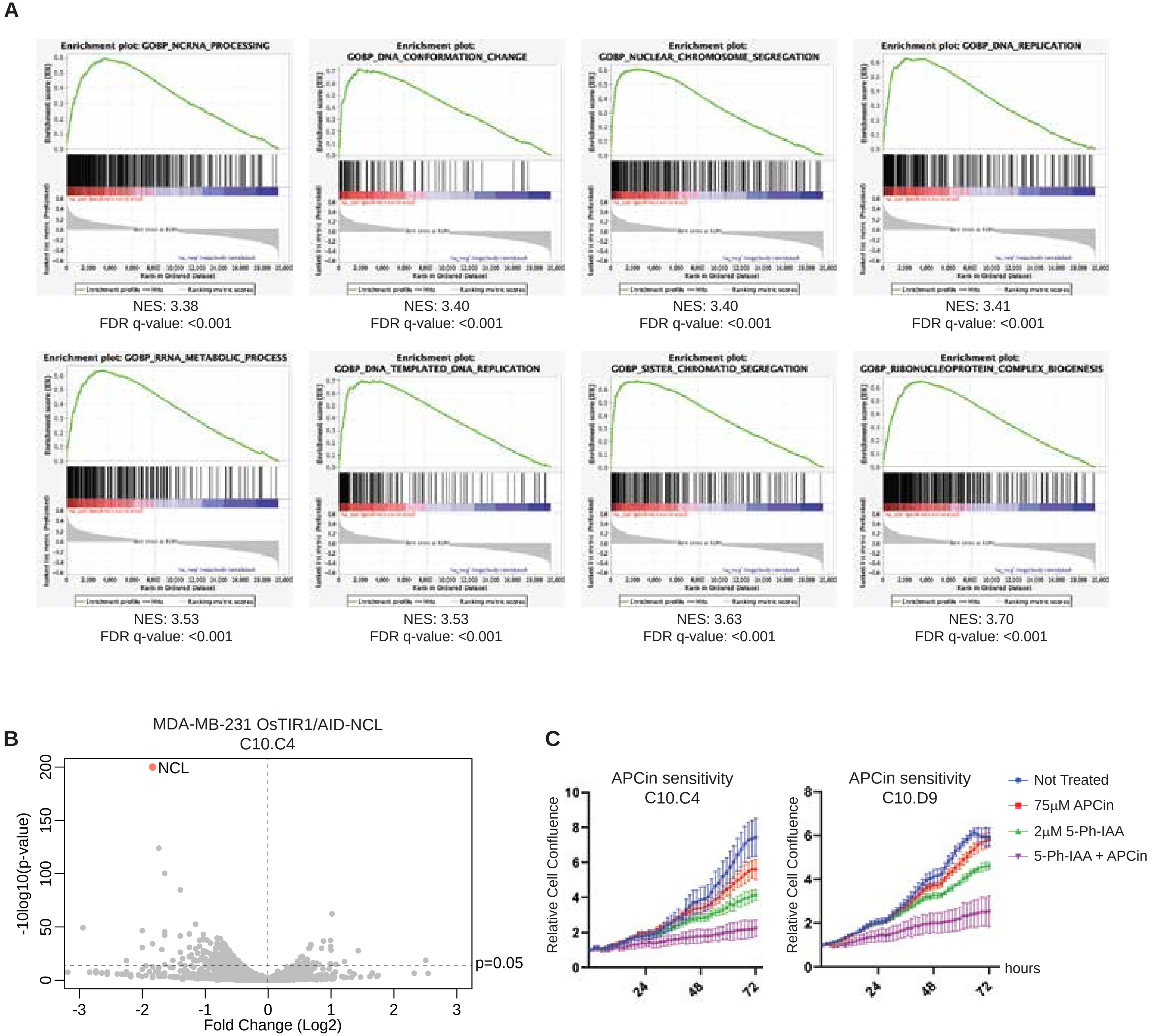
A
Enrichment plot: GOBP_NCRNA_PROCESSING
NES: 3.38
FDR q-value: <0.001
Enrichment plot: GOBP_DNA_CONFORMATION_CHANGE
NES: 3.40
FDR q-value: <0.001
Enrichment plot: GOBP_NUCLEAR_CHROMOSOME_SEGREGATION
NES: 3.40
FDR q-value: <0.001
Enrichment plot: GOBP_DNA_REPLICATION
NES: 3.41
FDR q-value: <0.001
Enrichment plot: GOBP_RRNA_METABOLIC_PROCESS
NES: 3.53
FDR q-value: <0.001
Enrichment plot: GOBP_DNA_TEMPLATED_DNA_REPLICATION
NES: 3.53
FDR q-value: <0.001
Enrichment plot: GOBP_SISTER_CHROMATID_SEGREGATION
NES: 3.63
FDR q-value: <0.001
Enrichment plot: GOBP_RIBONUCLEOPROTEIN_COMPLEX_BIOGENESIS
NES: 3.70
FDR q-value: <0.001
B
MDA-MB-231 OsTIR1/AID-NCL
C10.C4
NCL
-10log10(p-value)
0
50
100
150
200
p=0.05
-3
-2
-1
0
1
2
3
Fold Change (Log2)
C
APCin sensitivity
C10.C4
Relative Cell Confluence
0
2
4
6
8
10
24
48
72
APCin sensitivity
C10.D9
0
2
4
6
8
hours
Not Treated
75µM APCin
2µM 5-Ph-IAA
5-Ph-IAA + APCin

Raw Data

Figure 2D

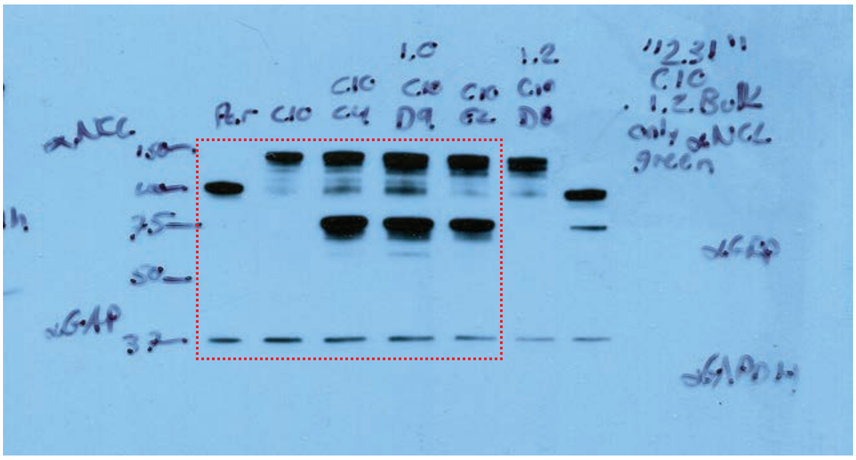

Figure 2E

NCL

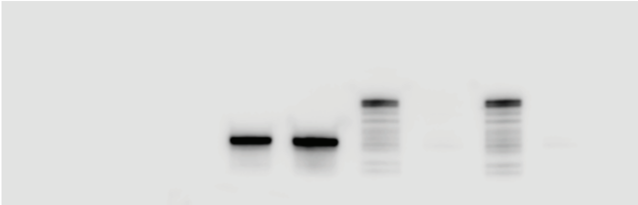

Calnexin Re-probed after NCL

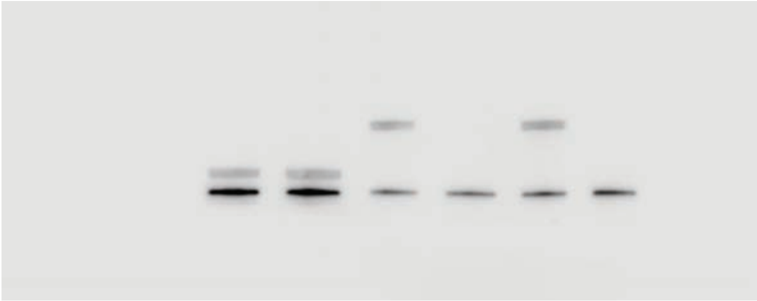

Figure S2F/G

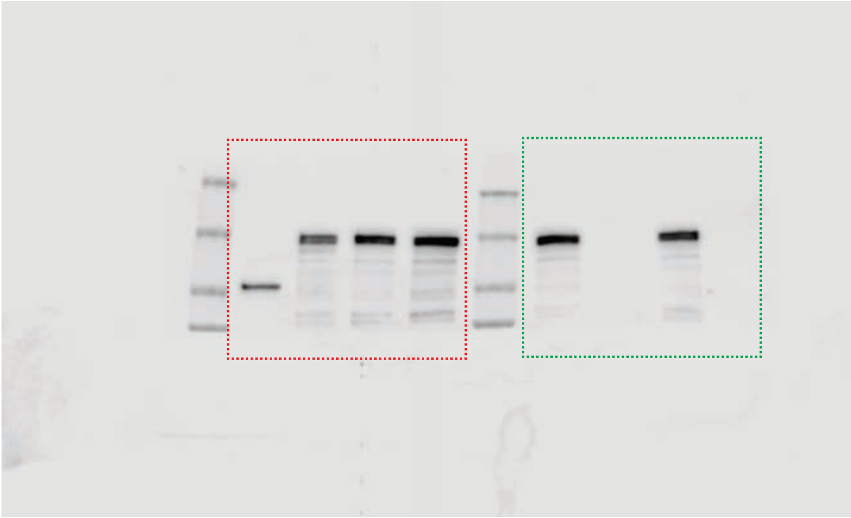

NCL

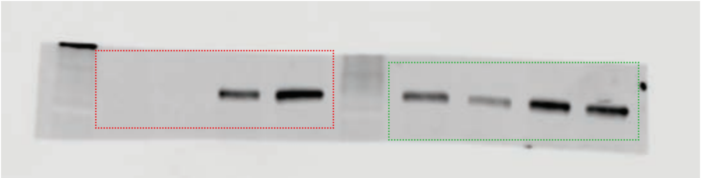

OsTIR1

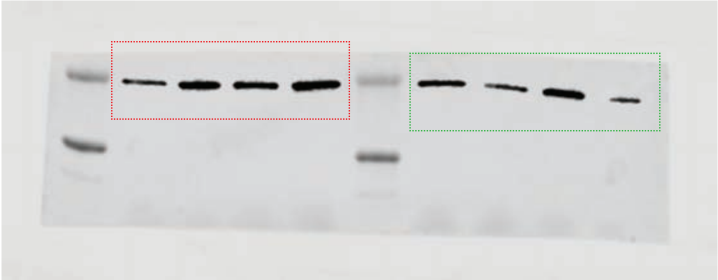

GAPDH
